# Iodine status of reproductive age women and their toddlers in northern Ghana improved through household supply of iodized salt and weekly indigenous meal consumption

**DOI:** 10.1371/journal.pone.0216931

**Published:** 2019-05-31

**Authors:** Clement Kubreziga Kubuga, Abdul-Razak Abizari, Won O. Song

**Affiliations:** 1 Nutritional Sciences Department, University for Development Studies, Tamale, Ghana; 2 Food Science and Human Nutrition Department, Michigan State University, East Lansing, MI, United States of America; Institut de recherche pour le developpement, FRANCE

## Abstract

Iodine deficiency (ID) during pregnancy results in pregnancy losses, intrauterine growth retardation, and lower IQ in the offspring. Even after two decades of universal salt iodization (USI) implementation, the efficacy of USI has not been reported in high risk groups in vulnerable regions in Ghana. We aimed to assess and improve ID status in childbearing age women (all lactating women) and their toddlers in northern Ghana, a geographically and socioeconomically vulnerable region. We provided weekly supply of household iodized salt and community-based feeding of native *Hibiscus Sabdariffaa* leaves meal (HSM) prepared with iodized salt to women and their toddlers in intervention (n = 60) vs. control group (n = 60). At baseline, ID was prevalent in women (36%) and their toddlers (29%). For women, both median UIC values for intervention (57.4 ug/l) and control group (65.1 ug/l) were below the recommended UIC value of 100 ug/l with no significant differences between the two groups (p = 0.2778). At the endpoint, median UIC for the intervention group (123.6 ug/l) was significantly higher (p = 0.008) than the control group (59.7 ug/l). Our results suggest that weekly supply of iodized salt along with the feeding HSM is an effective channel for improving iodine status of economically disadvantaged groups in communities remote from coastal lands. Furthermore, our results suggest that decreased median UIC among lactating mothers does not necessarily imply lower iodine status for their breastfed toddlers. And finally, the observed median UIC<100 ug/l may point to a non-improvement in iodine status for the past decade for Ghana. There is a need to revisit, assess, and ascertain the challenges in preventing populations from attaining the intended benefits of the USI policy in Ghana.

## Introduction

Iodine is an essential trace mineral for living organisms as an intramolecular component for the biosynthesis of thyroid hormones. Thyroid hormones control cell growth and differentiation, increase proteins, lipids, and carbohydrates metabolism [1]. Iodine deficiency disorders, a collective number of health outcomes, are the world’s leading causes of preventable cognitive retardation and poor psychomotor development in children [2, 3]. Iodine deficiency (ID) is commonly associated with goiter, intellectual impairments [4], growth retardation, neonatal hypothyroidism, and increased pregnancy loss and infant mortality [5, 6].

Iodine needs are mainly met from diet or supplementation. Major dietary sources of iodine vary from country to country dependent on soil content and food sources [7, 8]. Those major sources of dietary iodine in developed countries such as dairy products, eggs, seafoods and fortified foods are not readily available or consumed in adequate amounts in developing countries [9, 10]. Furthermore, feed for cows and chicken in developed countries are fortified with iodine [1]. The most important alternative source of iodine in developing countries, particularly in locations far from the ocean, is iodized salt [1, 11]. Iodized salt is the most common source of iodine for most African countries for which Ghana is no exception [12].

Globally, about 1.88 billion people have insufficient dietary iodine intakes with most of them living in economically disadvantaged areas [13] including Africa. In an effort to cope with the high magnitude of global burden of ID, World Health Organization (WHO)/United Nations Children’s Fund (UNICEF)/International Council for Control of Iodine Deficiency Disorders (ICCIDD) (nowadays called Iodine Global Network) recommended universal salt iodization (USI) [14]. Since 1994, USI is seen as a safe and cost-effective strategy to mitigate ID as salt is consumed by all individuals and in all countries. USI began in Ghana in 1996 upon passing a legislation to enforce iodization of salt (50 ppm and 25 ppm at points of production and retail respectively). WHO also recommend routine and regular monitor of a population’s iodine status [8, 14] by urinary iodine concentration (UIC). Since over 90% of ingested dietary iodine is excreted in the urine, the median UIC from spot urine samples is used in assessing iodine status of the population, but not that of an individual [8, 14, 15].

ID has recently been re-emerging as a public health concern, even in some industrialized countries such as Australia and the United Kingdom, which have not adopted USI and hitherto were regarded as iodine-sufficient countries [8, 16–20]. In developing countries, the burden of ID is further exacerbated [6, 13]. Currently in Ghana, ID prevalence among women of reproductive age is unknown as there is no data available on UIC. Per the 2014 National Demograhic Health Survey, household usage of iodized salt fortified at adequate concentration (≥15 ppm) was about 38.5% at the national level. The rate was much lower in inland regions of Northern (16%) and Upper East (18%). In general, households in rural areas use iodized salt at a much lower rate (26%) than those in the urban areas (50%) [21]. Lactating women and their toddlers have increased iodine needs and are vulnerable to iodine deficiency disorders [22, 23]. It is recommended that dietary intakes of iodine for toddlers and lactating women in areas of ID should be 15 ug/kg/day and 3.5 ug/kg/day, respectively compared to 4 ug/kg/day and 2 ug/kg/day for a 7–12 years old and adolescent/adult respectively [22]. Mother’s iodine status is very crucial source of meeting their infants’ iodine status via breast milk [23]. This study had two aims: 1) to investigate the prevalence of iodine status in women of reproductive age and their toddlers in the Upper East region in Ghana and 2) to investigate if the household iodized salt supply along with community-based feeding native *Hibiscus Sabdariffa* leaves meal (HSM) can improve iodine status of child-bearing age women and their toddlers (6-24mo) during dry/lean season. *Hibiscus Sabdarifa* leaves are commonly consumed staple vegetables especially in northern Ghana. Leaves are prepared a bit watery (as soup) and consumed with ‘tou zaafi’ (millet or corn based cooked paste). Leaves are also prepared thick (as a meal) and eaten by itself. In this study, the meal form was used. *Hibiscus Sabdarifa* leaves are eaten throughout the year and are in abundance in the rainy season.

## Subjects and methods

### Study design

The design of this study was a quasi-experimental, community-based, 12-week feeding trial (clinical trails.gov ID: NCT03754998) with the primary focus of this paper being iodine status of mother-child dyads, determined by urinary iodine concentration (UIC). At the time of planning the current community-based intervention study (2015/2016), PIs did not see the study fit under the classical definition of clinical trials that include any novel products (drugs, foods, instruments), but the definition has expanded since.

### Study site

**Fig 1:**
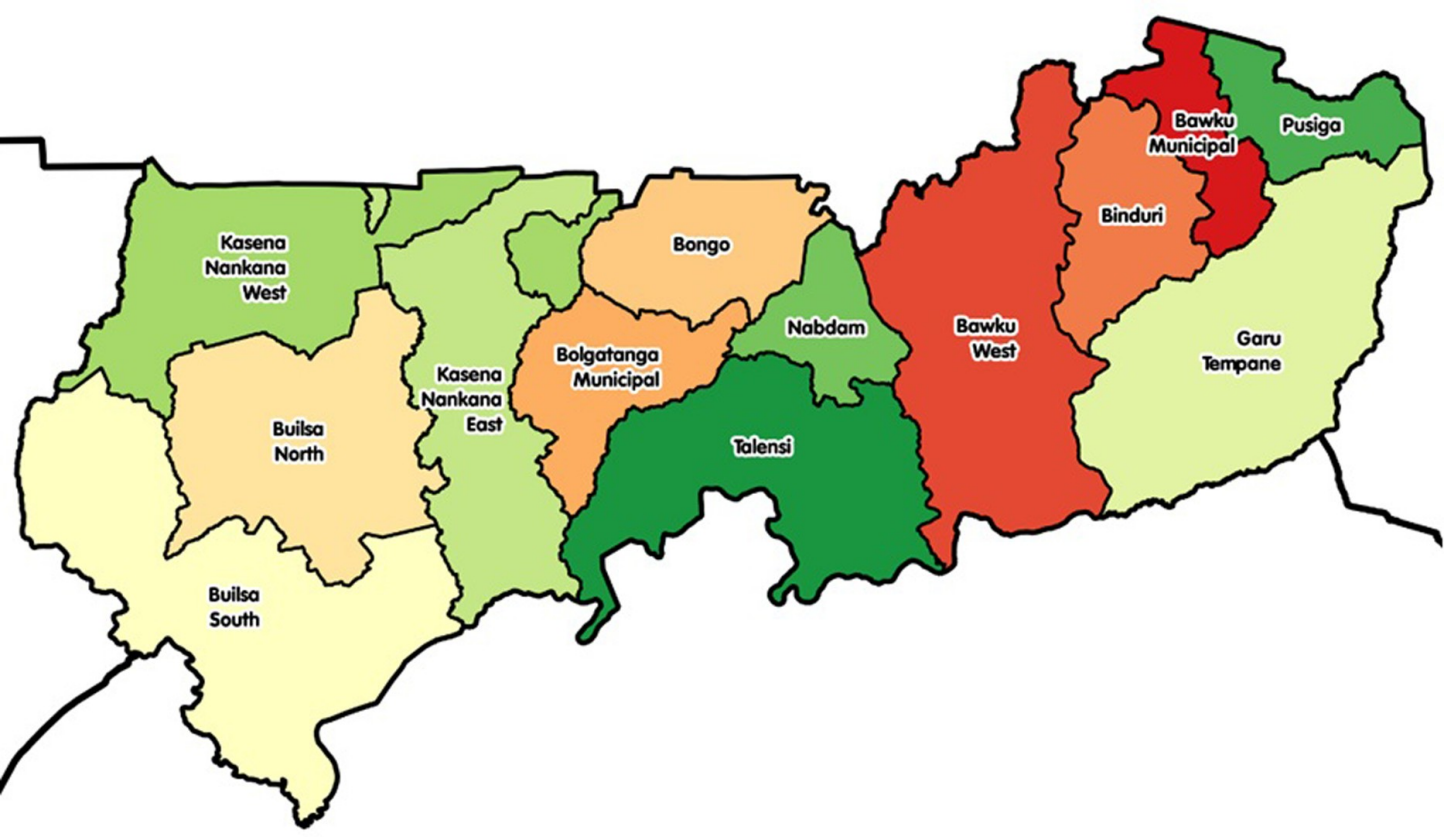
A map of Upper East region showing its districts.

This study was carried out in two districts in Upper East Region in Ghana: Kassena Nankana West (KNWD) and Builsa North (BND) districts (Fig 1). The study sites were narrowed down to two communities (Sakaa and Chania) in KNWD and three communities (Chuchuliga-yipaala, Azoayeri, and Awulansa) in BND. These districts were among the top five food insecure districts in the region. Sites were selected based on the inclusion criteria of having a functional borehole (water source) throughout the dry season, existing women groups, and access to Community-Based Health Planning and Services (CHPS) compounds, sizeable number of mother and young children (6–23 mo) dyads for good sampling frames and community health nurses who were willing to work with researchers between May and August 2016. Researchers’ previous experience with those communities facilitated the community entry process.

### Study subjects and selection

We recruited women who were between 15–49 yrs, had a child (6–23 months), a member of an existing community-based women group or willing to join one in their respective communities, willing to participate and available throughout our study period (May-September, 2016). These women were part of a feeding trial study as well as a dry season container garden project.

The dyads were drawn from a pooled list of selected districts using community-based birth registers at community health centers kept by community health nurses stationed in these communities or by community health volunteers. Health volunteers are community members. Announcements through the community chiefs/leaders were then made in the respective communities for all women with under 5 years of age to meet at their respective community health centers.

Research team briefed the women and the community leaders on the study. Names that were shortlisted from the birth registers were read out. Dyads were contacted individually (including dyads whose names were not in register but were obtained from the health volunteers who knew almost everybody in their catchment areas) to check on their willingness to be part of the study through the community health volunteers and community health nurses. Finally, verbal consent was sought from spouses of the women who were willing to be part of the study.

**Fig 2:**
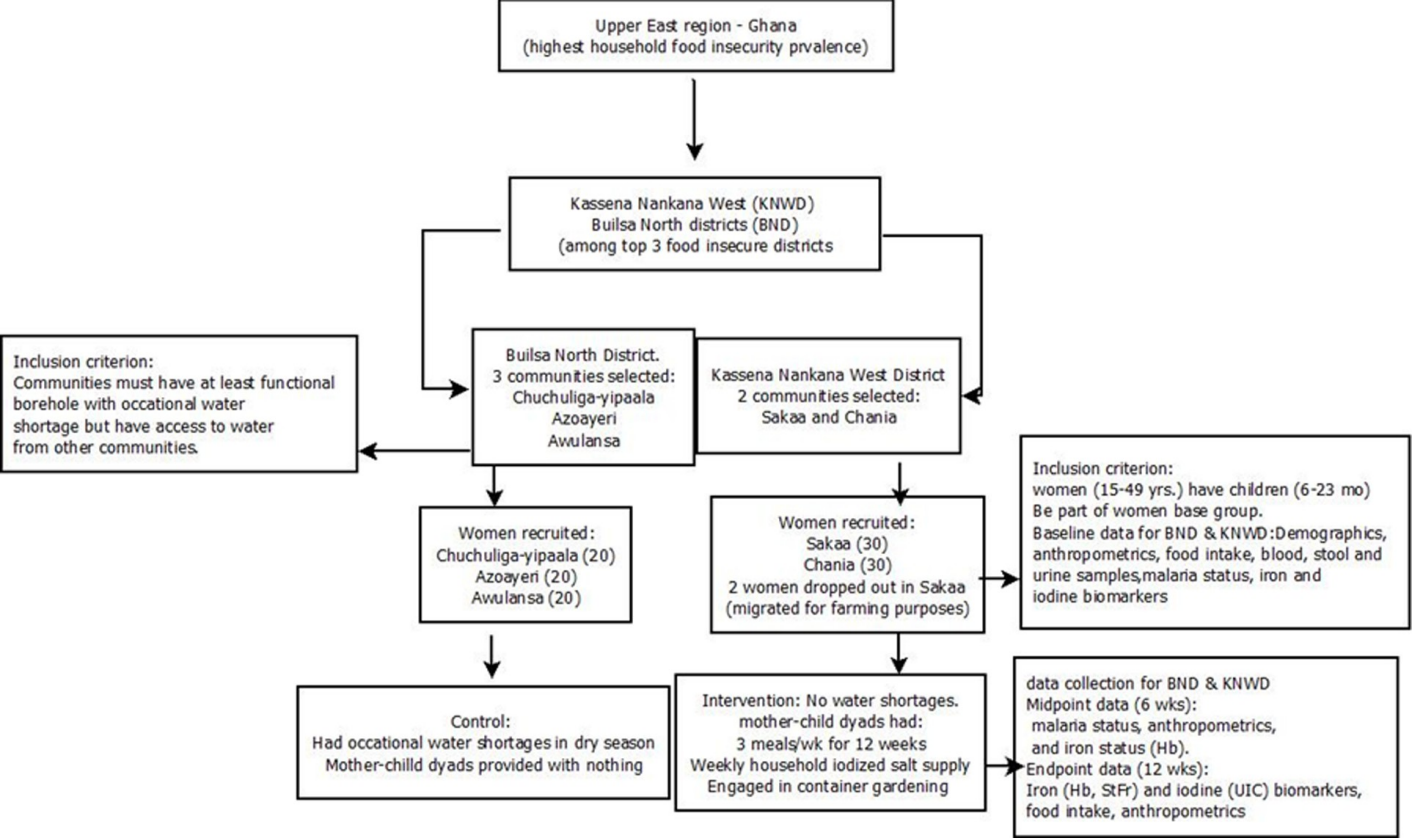
Study design.

A total of 120 dyads (Intervention—60 mother/child dyads; control—60 mother/child dyads) drawn from the two districts agreed to participate in the study (Fig 2). The intervention group was assigned at the community level to avoid contamination of the information at the individual level within a community. During the study, four dyads in intervention group relocated: two before baseline data collection and two in 6^th^ week of the study. All relocations were either to join a spouse or the entire nuclear family migrated for farming purposes. There was no replacement made in these instances.

### Intervention

The participating dyads in intervention communities were provided weekly supply of iodized salt (45 g) for household use, participated in a feeding trial with veo soup/meal three times a week, and dry season container gardening. The veo soup/meal is a local Ghanaian soup/meal mainly made of *Hibiscus Sabdariffa* leaves. Our standardized recipe for the meal of 52.5 kg contained *Hibiscus sabdariffa* leaves (18 kg), groundnut (8 kg), dawadawa (1.1 kg), dried fish (3 kg) and iodized salt (0.045 kg). In the standardized meal, the main source of iodine is the iodized salt. The meal was used to ensure compliance in consumption of iodized salt. Each dyad was given two separate bowls for mother and toddler to monitor their intakes to individual satisfaction. The women and toddlers consumed an average of 1.9 kg and 0.4 kg respectively per each meal. These amounts would contain 1.6 g and 0.3 g iodized salt for women and toddlers respectively. On feeding days, the meal was served (1.5 kg/woman) to all women and their children (0.5 kg/child) separately. The women and children were given two separate bowls, so that the researcher could monitor each person’s consumption. All dyads were encouraged to consume to their satisfaction by requesting additional servings. The meal intake was measured by the researcher and trained women by the differences between the quantities served minus leftovers. Details of the meal and community-based feeding has been published earlier [24].

### Measurements and data collection

Approval for the study was obtained on March 8, 2016 and May 2, 2016 from NHRC IRB in Ghana and MSU IRB respectively. The study was open for recruitment from May 20–27, 2016 followed by baseline data collection on May 30 –June 3, 2016. Intervention commenced on June 6, 2016, midpoint data excluding UIC was taken on July 23–24, treatment (feeding trial) ended on August 29, 2016. Final biomarker data collected on September 8, 2016 while data on container garden was taken on September 29. The authors confirm that all ongoing and related trials for this intervention are registered.

#### Questionnaire/interview

At baseline, information on sociodemographic characteristics and food intake was collected. Each subject was asked to bring a sample of salt used at her home for a rapid test for the presence of iodine. Content of iodine (0 ppm, <15 ppm, and ≥15 ppm) in household salt samples provided by women were tested using validated Rapid Test Kits (Batch No: M 050, MBI KITS International, India) according to prescribed procedures. Change of color that indicates iodine concentration was compared with the RTK charts and recorded. Spot urine samples of participants were taken in labeled plastic bottles at baseline (0 wks) and at endpoint (12 wks). Investigations were carried out following the rules of the Declaration of Helsinki of 1975. Ethical approval was obtained from Michigan State University’s Ethical Review Board–MSU IRB (May 2, 2016) as well as Navrongo Health Research Center’s (NHRC) IRB in Ghana (March 8, 2016).

#### Anthropometry

Participants’ weights and heights were measured at baseline, at 6 wks and at 12 wks according to standard procedures [25]. Electronic scale (UNIscale; Seca) was used to measure weights to the nearest 0.1 kg. The scale was calibrated using a known weight on the days when measurements were taken.

#### Iodine status assessment

WHO provides two criteria for assessing iodine status in populations: use of median UIC of spot urine samples and proportion of the total healthy population with UIC below and above 50 ug/l. The median UIC of <100 ug/l in the child-bearing age lactating women and their toddlers <2 yrs, iodine concentration indicates inadequate iodine intake with no other categories of iodine intake defined [14]. More than 20% of population with UIC <50 ug/l indicates inadequate iodine intake of the population investigated. We assessed the iodine status of the study population using median UICs and also by the prevalence of <50 ug/l UIC. UIC was analyzed using the Sandell-Kolthoff (acid-digestion) reaction [26, 27] and results were expressed as micrograms of iodine per liter. The inter-assay CV in the laboratory is 6%. Urine samples were held at ambient temperature on the field and during transportation to NHRC. The samples were stored at −18°C at NHRC for four months and then transported on dry ice to Noguchi laboratory for UIC analysis. The lab is certified for its quality control by the CDC’s EQUIP program.

#### Covariates

The study subjects were reproductive age women (15–49 yrs) with their toddlers (6–23 mo). Sociodemographic and behavioral variables included household usage of iodized salt, age of woman (yrs), height of woman (cm), education of women, occupation of women, ethnicity, religion, household head sex, household head’s age, wealth index [28], number of household adults, age of toddlers, and number of children < 5 yrs.

### Statistical analysis

Data analyses were conducted using SAS (version 9.4, SAS Institute Inc., Cary, NC). Characteristics of participants were described using frequency distributions. Comparison of participants’ characteristics between the intervention vs. control groups was done using chi-square statistics and Student t-test for categorical and continuous variables, respectively. We estimated and compared median UICs of both groups. Additionally, the prevalence of <50 ug/L UIC between intervention and control groups were estimated to assess iodine status in relation to treatment.

For the study sample size, optimal design software (version 3.01) was used to calculate the minimal sample size of 100 dyads (50 dyads each for intervention and control groups) with a power of 80%, significance level of 5%, coefficient of determination of 65%, minimum detectable effect of 0.33, and 20% attrition rate. reference for formulae used by this software can be found online [29].

## Results

The subjects of this study were 118 women (15–49 yrs) and their toddlers (6–23 mo) dyads. We had missing UIC values (baseline:n = 52 endpoint: n = 16) due to samples not having sufficient volume for analysis or containers being empty upon arrival at the Noguchi Iodine Laboratory-Ghana. We thus checked to see if these missing values were evenly distributed in both groups using the χ^2^ test (baseline: χ^2^ = 1.60, P = 0.2057; endpoint: χ2 = 1.14, P = 0.7130) for women’s data. We further checked to see whether individuals with missing UIC had different characteristics (wealth index and education) as compared to those without missing data. Wealth index and education were of particular interest as they are key determinants of iodized salt use in other studies. For toddlers, missing values were also evenly distributed in the baseline and endpoint data for control and intervention groups.

Sociodemographic characteristics and UIC status of the participants are summarized in Tables 1 and 2. At the baseline, the intervention group did not differ significantly from the control group, except for occupation (p = 0.0007), religion (p<0.0001), ethnicity (p<0.0001) of women, household iodized salt usage (p = 0.002), iodine level in household salt (p = 0.019), and UIC<50 ug/l in toddlers (p = 0.021). The majority of households (81% of intervention and 98% of control group) did not use iodized salt (0 ppm). On adequate consumption of iodine (salt iodine content ≥15ppm), 8.8% and 0.0% of households consumed salt with adequate iodine amounts in intervention and control groups respectively at baseline. In our study population, 44.8% and 29.7% of the women had UIC< 50 ug/l in intervention and control groups, respectively (Table 2). Among the toddlers, 15.6% and 41.2% had UIC<50 ug/l in intervention and control groups, respectively. There was no significant difference in the proportion of women with UIC<50 ug/l between groups at baseline (p = 0.2057)

**Table 1:** Demographic characteristics of child-bearing age women (15–49 yrs) and their toddlers (6–23 mo) in intervention and control groups at baseline.

Variable		Intervention		Control		P-value
		n	Mean ± SD	n	Mean ± SD	
Women age (yrs)		58	26.7±6.7	60	26.1±5.4	0.598
Toddlers age (months)		59	14.1±5.1	62	12.6±5.2	0.119
Household head’s age		58	47.0±16.9	60	42.7±15.1	0.149
# of adults in household		58	4.6±3.3	60	3.7±1.4	0.051
# Siblings in household		59	1.4±1.6	62	1.9±1.5	0.092
# children < 5 yrs		58	1.0±1.2	60	1.1±1.2	0.708
Variable		n	%	n	%	p-value
Occupation of women	Farmer	31	53.5	50	83.3	0.002
	Handy works	5	8.6	5	8.3	
	Housewife	6	10.3	2	3.3	
	Trader	16	27.6	3	5.0	
Household head sex	Male	54	93.1	56	93.3	0.960
	Female	4	6.9	4	3.4	
Wealth index	Lower	19	32.8	20	33.3	0.991
	Middle	20	34.5	20	33.3	
	Upper	19	32.7	20	33.3	
Decision making	Low	11	20.0	9	15.0	0.545
	Average	27	46.6	34	56.7	
	Above	20	34.5	17	28.3	
Education	None	19	33.3	14	23.3	0.486
	Primary	18	31.6	22	36.7	
	Above primary	20	35.1	24	40.0	
Household iodize salt	Non-users	46	80.7	59	98.3	0.002
	Users	11	19.3	1	1.7	
Iodine level in household salt	<15ppm	52	91.2	60	100.0	0.019
	≥15ppm	5	8.8	0	0.0	
Ethnicity of women	Builsa	2	3.4	56	90.3	< .000
	Kassena	55	93.2	5	8.1	
	Nakani/Fulani	2	3.4	1	1.6	
Religion	Christian	34	57.6	60	96.8	< .000
	Muslim	9	15.3	0	0.0	
	Traditionalist	16	27.1	2	3.2	

Traditionalist: Practitioners of African Traditional Religion; Decision making: the degree in which women participate in household decision making. Wealth index: Household cumulative living standard measured by using household assets

**Table 2:** Baseline health indicators of child bearing age women (15–49 yrs) and their toddlers (6–23 mo) in intervention and control groups.

		Intervention			Control			
Variable		n	Mean± SD	Median	n	Mean± SD	Median	P-value
**WOMEN**								
Height (cm)		58	160.3±6.6		60	161.7±5.6		0.217
Weight (kg)		58	57.1±9.7		60	57.3±7.6		0.888
BMI		58	22.2±3.6		60	21.9±2.3		0.537
UIC,Median (ug/l)		29		57.4	37		65.1	
**TODDLERS**								
HAZ		59	0.3±1.3		62	-0.3±1.3		0.009
WHZ		59	-1.2±1.3		62	-0.9±1.3		0.294
UIC,Median (ug/l)		32		150.2	34		90.4	
		n	%		n	%		p-value
**WOMEN**								
UIC (ug/l)	<50	13	44.8		11	29.7		
	≥50	16	55.2		26	70.3		0.205
**TODDLERS**								
UIC (ug/l)	<50	5	15.6		14	41.2		
	≥50	27	84.4		20	58.8		0.021
HAZ	>-2SD	3	5.08		7	11.29		
	<-2SD	56	94.92		55	88.71		0.215

UIC: Urinary iodine concentration; Iodine deficiency defined by UIC <50 ug/l. HAZ: height-for-age z-score;

At the endpoint, there was a significant difference (p = 0.0073) in the proportion of women with UIC<50 ug/l between groups (Table 3). In toddlers, there was a significant difference between the intervention vs. control groups in the proportion of those with UIC<50 ug/l at baseline (p = 0.0219) but not at endpoint (0.0670).

**Table 3:** Median UIC and distribution of UIC of childbearing age women (15–49 yrs) and their toddlers (6–23 mo) in both intervention and control groups across time.

Time line	Status		Intervention					Control				χ^2^	^+^P-Value
		n	%	Median	95% Median CI	P-value*	N	%	Median	95% Median CI	P-value*		
**Women**													
Baseline	UIC ug/l	29	43.9	57.4	(36.9,75.8)	<0.0001	37	56.1	65.1	(58.3,79.5)	0.172		0.085
Endpoint		49	48.0	123.6	(76.7,231.2)		53	52.0	59.7	(52.3,71.7)			0.001
Baseline	UIC<50 ug/l	13	44.8			<0.0001	11	29.7			0.842	1.6	0.206
	UIC≥50 ug/l	16	55.2				26	70.3					
Endpoint	UIC<50 ug/l	5	10.2				17	32.1				7.2	0.007
	UIC≥50 ug/l	44	89.8				36	67.9					
**Toddlers**													
Baseline	UIC ug/l	32	48.5	150.2	(84.4,406.2)	0.0084	34	51.5	90.4	(34.6,179.6)	0.379		0.1425
Endpoint		29	54.7	198.0	(137.7,324.4)		24	45.3	104.1	(60.8,141.0)			0.009
Baseline	UIC<50 ug/l	5	15.6			0.2708	14	41.2			0.161	5.2	0.0219
	UIC≥50 ug/l	27	84.4				20	58.8					
Endpoint	UIC<50 ug/l	2	6.9				6	25.0				3.4	0.067
	UIC≥50 ug/l	27	93.1				18	75					

UIC: Urinary iodine concentration,

χ^2^: Chi–square test,

UIC<50 ug/l: *inadequate iodine status*,

*P-value: within group comparison for chi-square test,

^+^P-value: between groups comparison for chi-square test

At baseline for women, both median UIC values for intervention (57.4 ug/l) and control group (65.1 ug/l) were below the recommended UIC value of 100 ug/l with no significant differences between the two groups (p = 0.2778). At the endpoint, median UIC for the intervention group (123.6 ug/l) was significantly higher (p = 0.008) than the control group (59.7 ug/l). Similarly, in toddlers, no significant differences were in the median UICs between the two groups (p = 0.1425) at baseline but a significant difference observed at the endpoint (p = 0.009). Toddlers in both groups had median UIC values higher than their mothers. However, median UIC values in both the control and intervention groups experienced an increment with time.

## Discussion

To the best of our knowledge, this is the first interventional study that assessed iodine status of women and their toddlers in northern Ghana. Uniquely we utilized an indigenous Ghanaian meal as a vehicle for salt intake compliance to improve iodine status of childbearing age women (lactating women) and their toddlers 6-24mo dyads in northern Ghana. Several studies have focused on the prevalence of iodine deficiencies among women and children [30–32].

We are alarmed to find that most of the households consuming non-iodized salt at baseline: intervention (81%) and control (98%) in comparison with the reported national average (66%). Furthermore, salt samples from all households in intervention (91%) and control (100%) groups contained inadequate concentration of iodine in salt, suggesting that USI program in northern Ghana has not been successfully implemented to date. In this regard, it is not surprising that the women and toddlers had severe ID. Salts with 0ppm or inadequate iodine partly come from small scale salt miners. Additionally, we cannot preclude the possibility that iodine in salt might have been lost prior to purchase at the store or storage at households [33] under extremely wet and hot seasons. On the contrary, 55% and 70% of the women had their UIC>50 ug/l). This may point to alternative iodine sources consumption [34]. This may raise a question as to whether iodized salt is still a key source of iodine for households in the inter lands. This legitimate question partially contributed to the conduction of our intervention study.

We found that the weekly household iodized salt supply (450 g) and additional consumption of iodized salt (48.9 g/wk/adult and 10.2 g/wk/child) using HSM as a compliance channel significantly improved the iodine status of women in the intervention group. There was a significant increase in proportion of participants with UIC ≥50 ug/l in intervention group while a decline was observed in the control group (p = 0.021). Median UIC of the intervention group was also significantly higher than that of the control group. Though women had non-optimal iodine status, iodine status of their toddlers was within or near optimal levels. A possible biological explanation for this observation could be due to compensatory mechanism in the mammary glands to provide iodine enriched milk for children [35]. Though toddlers had higher iodine status, it appears the compensatory mechanism in the mammary glands strives to keep milk iodine status within or near optimal levels [23]. Our findings indicate that decreased urinary iodine levels among lactating mothers does not necessarily imply lower iodine status for their breastfed toddlers considering the observations in median UIC of the toddlers.

Our findings further suggest that iodized salt is a key source of iodine in the research settings. Additionally, it is an effective channel for the improvement of iodine status of economically disadvantaged groups living in remote areas in vulnerable locations due to geographical distance from coastal lands [13]. Our findings are in consonance with other clinical trial studies [36] that show a positive improvement of median UIC by iodized salt use in toddlers. A recent study published by Nazeri and colleagues [35] reported beneficial effect of iodine fortified foods in improving the median UIC of lactating women. Another study in northern Ghana [34] reports the beneficial effect of iodine fortified bouillon cubes. The study further suggests that the bouillon cubes were the major source of iodine intake among school children and that iodine adequacy was largely due to the bouillon cubes but not iodized salt. Their finding is contrary to what is observed in our control group. If bouillon cubes were a major source of iodine, decline in median UIC might have been unlikely.

According to our results, ID in the region is within the classification for Ghana being among countries with mild ID per WHO classification (Median UIC of 50–99 ug/l) for the period of 1993–2006 [37–39]. While our findings (Median UIC<100 ug/l) among women is worrisome, improvement in median UIC is expected as a result of Ghana passing legislation to implement the universal salt iodization policy in 1996 [40]. As there has been no improvement for a decade, this could be an indication of the ineffectiveness of policies and programs addressing iodine intake and iodine adequacy in the population for the last decade. This assertion is further corroborated by the high proportion of households (82%) not using adequately iodine fortified salt in the region. We suggest that activities and programs aligned with universal salt iodization should be revisited and assessed to ascertain the bottlenecks preventing the populations from attaining the intended benefits of the universal salt iodization adopted by Ghana.

This study is not without limitations and strengths. The strengths of our study include mainly an intervention study with a control group for comparison, urinary iodine concentration measurements taken at baseline and endpoint. Using an indigenous meal to ensure compliance of iodine intake is the first of its kind in the research settings and further strengthen our findings. We recognized that not being able to estimate dietary iodine intakes could be a drawback which have not been able to overcome in any studies to date. However, iodized salt is apparently the main source of dietary iodine in northern Ghana. Our results are limited to population level interpretation as we recognized the limitation of a single spot UIC [41] for an individual’s iodine status.

## Conclusion

To the best of our knowledge, this is the first interventional study that assessed iodine status of women and their toddlers in northern Ghana, utilized an indigenous Ghanaian meal as a vehicle for salt intake compliance to improve iodine status of childbearing age women (lactating women) and their toddlers 6-23mo dyads. We found a high prevalence of severe ID (>20%). Our results suggest that iodized salt is an effective channel for the improvement of iodine status of economically disadvantaged groups living in remote areas in vulnerable locations due to geographical distance from coastal lands. Our findings further suggest that decreased urinary iodine levels (median UIC) among lactating mothers does not necessarily imply lower iodine status of their breastfed toddlers. Further research is needed to ascertain the bottlenecks and challenges preventing populations from attaining the intended benefits of the universal salt iodization policy adopted by Ghana over two decades ago. Efficacy of USI has to be monitored because many factors such as cost, inadequate level of fortification, or loss of iodine during storage can alter the expected outcome in prevention of ID in public.

## Supporting information

S1 File Protocol. (PDF)

S2 File Trend statement checklist. (DOC)

S3 File Appendix F–Questionnaire. (DOCX)
