# Supplementary material for: Iodine status of reproductive age women and their toddlers in northern Ghana improved through household supply of iodized salt and weekly indigenous meal consumption
Source: PLoS One. 2019 May 31;14(5):e0216931. doi: 10.1371/journal.pone.0216931 (PMC6544231; doi:10.1371/journal.pone.0216931)
Supplement: S1 File — (PDF) [file pone.0216931.s001.pdf]

Community interventions to improve iron and iodine status in mother and child dyads in northern Ghana

PhD Dissertation Proposal

By
Clement Kubreziga Kubuga

Advisors:
(US) Won O. Song, PhD, MPH, RD
(Gahna) Abdul-Razak Abizari, PhD
(CGIAR) Gina Kennedy, PhD

Guidance Committee Members

Dr. Sarah Comstock
Dr. Andrew Dillon
Dr. Won Song (Advisor)
Dr. Lorraine Weatherspoon

Guidance Committee Meetings

1st Committee Meeting – 5/1/15
2nd Committee Meeting – 3/4/16
3rd Committee Meeting – 4/22/16

Michigan State University

Contents

LIST OF TABLES **3**
LIST OF FIGURES **4**
LIST OF ABBREVIATIONS AND ACRONYMS **5**
CHAPTER 1: INTRODUCTION **6**
1.1 Background 9
1.2 Study aims and hypothesis 10
1.3 Significance 11
CHAPTER 2: LITERATURE REVIEW **13**
2.1 Food security vs nutrition security 13
2.2 Micronutrients in human health 14
2.3 Dietary diversity score as a measure of diet quality 18
2.4 Interventions to improve trace mineral status with dietary approaches 23
2.5 Dietary diversity score as a measure of disease outcome 24
2.6 Interventions to improve trace mineral status with dietary approaches 24
2.7 Household food production to improve nutritional status 28
CHAPTER 3: METHODS AND APPROACHES **29**
3.1 Aim1 - Screening tool development 31
3.2 Aim 2 – Feeding trial 35
3.3 Aim 3 – Container gardening 37
3.4 Study ethics 39
REFERENCES **41**
APPENDICES **46**
Appendix A: Nutrient composition of staple foods in the Upper East Region of Ghana 46
Appendix B. Dyad Consent Form-Intervention 47
Appendix C: Dyad Consent Form-Control 50
Appendix D: Parental Consent Form-Intervention 53
Appendix E: Parental Consent Form-Control 56
Appendix F: Questionnaire 59
Appendix G: MSU IRB Form 64
Appendix H: Ghana nutrition health and population statistics 65
Appendix I: Budget 70

LIST OF TABLES

Table 1: Dietary diversity score as a measure of diet quality... 21

Table 2: Dietary base interventions to improve nutrients intake... 26

LIST OF FIGURES

Figure 1: Research goal and aims 12

Figure 2: Study frame 30

Figure 3: Aim 1 and its research approaches 31

Figure 4: Aim 2 and its research approaches 35

Figure 5: Aim 1 and its research approaches 37

LIST OF ABBREVIATIONS AND ACRONYMS

DDS - Dietary Diversity Score Dietary

DHS – Demographic and Health Survey

FAO - Food and Agriculture Organization

FVS - Food Variety Score

GSS – Ghana Statistical Service

HDDS – Household Dietary Diversity Score

IFPRI - International Food Policy Research Institute

CHAPTER 1: INTRODUCTION

Ghana is a lower middle income country among Sub-Saharan countries with less than 9% of its population reported being food insecure in 2011-2013 (FAO, 2015). However, the prevalence of malnutrition in mothers and young children has been persisting at alarmingly high rates (GSS, 2009): stunting rates in < children 5 years of age ranging from 34% (1999) to 23% (2011); iron and vitamin A deficiencies at the rates of 78%-76% in children and 59%-18% in women of 15-49 years of age (GSS, 2009; GSS, 2011); only 35% of households reported using iodized salt (GSS, 2011).

Devastating disparity exists in the rates of nutritional insecurity and stunting between the three northern regions and the remaining seven regions of Ghana (GSS, 2009; WFP, 2015). The disparity is evidenced by the rate of the poor at 60% in the northern regions vs. 20% in southern Ghana (WFP, 2012; 2015; World Bank, 2013). This is partially due to differences in climate, agriculture and food systems with limited arable farm land, food and cultural practices. The disparity is worse during the prolonged dry season for 7-8 months in northern Ghana when staple nutrient-rich vegetables (e.g. Hibiscus sabdarifa) and legumes/pulse are limited. This has a devastating effect on nutritional security and creates a cycle of food insecurity as cereals, vegetables, and legumes/pulses are the main staples of northern Ghana (Appendix A).

The vicious cycle that lead to malnutrition, and stunting in mothers and young children are exacerbated by trace mineral deficiencies. Numerous costly national and international initiatives such as National Vitamin A supplementation for children under 5yrs, pregnant and lactating women; universal salt iodization policy; iron and folic acid

supplementations to pregnant women (David, 2003; Saaka, 2012; Nyumuah et al., 2012; Saaka, 2012) have been introduced to counteract trace mineral deficiencies. Furthermore, efficacies of these programs at the national levels have not yet been reported. Iron and iodine deficiencies lead to fetal and infant growth retardation, stunting and impaired cognitive development. Mild iodine deficiencies seen in developed countries (e.g., England, Australia) are documented to result in lower IQs in children (Bath et al., 2013).

Nutritional deficiencies can be most efficiently assessed, screened and intervened by non-invasive dietary intake compared to costly and laborious biomarkers or non-specific clinical examinations. Currently there are no dietary assessment tools that are validated by biomarkers of trace minerals in Ghana, most importantly due to lack of food composition tables of indigenous foods. We aim at contributing to solving the aforementioned problem.

Stunting is resulted by prolonged nutrition insecurity that is dependent on adequate dietary intake. Stunting impacts one’s health status and productivity throughout his/her life. Prolonged inadequate diets imply inadequate intake of such important nutrients as trace minerals. It is well noted that stunting rates are also affected by several such factors as social, political, community, household, and individual factors (Stewart et al., 2013). The stable political environment, relative economic growth and improved health system over the years in Ghana (IFPRI, 2014) has been expected to reduce the stunting prevalence from what was being observed.

At an individual level, stunting is also resulted by limited catch-up growth in infants born with symmetric and asymmetric intrauterine growth retardation (IUGR)

(Strauss and Dietz, 1997). Several studies showed that IUGR is largely influenced by maternal deficiency of such trace minerals as iodine, iron, zinc and selenium (Caulfield et al., 2006; Strauss and Dietz, 1997) and macronutrients (Strauss and Dietz, 1997). Stunting is also resulted when children at the critical stages of growth are exposed to nutritional deficiencies of such trace minerals as iodine, iron, zinc and selenium (Kennedy et al., 2003). However the magnitude of deficiencies of these nutrients in children, except for anemia, have neither been monitored nor reported in Ghana. Detecting deficiencies of iron, iodine and zinc at the early stages are important because of their critically important biological roles in human health and growth (Allen et al., 2006). In field studies, biological detection of iron deficiency is commonly through measurements of hemoglobin concentration, iodine status by urinary iodine concentration and zinc status by serum zinc or urinary zinc concentrations. These laboratory-based biochemical measurements are however time-consuming and costly compared to dietary assessment, if dietary approaches can predict the biomarkers.

In developing countries, dietary assessment of trace minerals is challenging and limited due to incomplete or absence of food composition datasets of native regional foods. Dietary diversity score (DDS) has been the only known and widely used dietary assessment approach in developing countries to measuring food security and diet quality (Ruel, 2003a). Little is known on the association between different calculations of DDS and nutritional status of problematic nutrients as determined by their biomarkers and stunting in Ghana. Dietary patterns (DP) has also been suggested as an approach to predict the association between diet and development of diseases (Hoffmann et al., 2004). The rationale of the suggestion is that foods are consumed in combination

making it difficult to separate the effects of single foods on the development of diseases in observational studies (Hoffmann et al., 2004). To our best knowledge, to date no Ghanaian diets have been investigated for their associations with trace minerals biomarkers. DDS and DP may serve as easier, quicker and noninvasive approaches to predicting population based trace mineral deficiencies for subsequent interventions in a long term. In the short term we seek to investigate dyads trace mineral status (iron and iodine) with household characteristics, consumption, and access to vegetables, iodized salt, and high quality protein (fish) in dry/lean season through a demonstration project.

1.1 Background

Ghana has reduced food insecurity prevalence by 49.2% in two decades. However, prevalence of malnutrition especially stunting (in <children5yr) has not matched the changes in food security levels. Of several key nutritional factors that lead to stunting, nutritional status of such trace minerals as iodine, iron and zinc has not been studied. Nutritional inadequacy of these trace minerals may be detected by estimation of dietary intake, and time-consuming and costly biochemical measurements of respective biomarkers. Since there is no means to estimate dietary intake of the trace minerals in Ghana, due to incomplete food composition data of the nutrients, identifying validated non-invasive, dietary approaches to predict the biomarker status of these trace minerals are critical in counteracting the challenges surrounding the persistent stunting due to micronutrient deficiencies in Ghana. Additionally, exploring alternative approaches to providing access to foods rich in trace minerals at household level is crucial.

1.2 Study aims and hypothesis

The ultimate goal of this research is to increase knowledge base on improving trace mineral status in mother-child (6-23 mo) dyads through a sustainable community-based interventions in northern Ghana. We will begin with iron and iodine that impair mothers and young children's growth and cognitive development most with three aims: 1) to develop dietary screening tools that are validated by biomarkers for early detection of deficiencies, 2) to determine efficacy (dose responses) of feeding indigenous nutrient-rich meals in preventing deficiencies and improving iron and iodine status, and 3) to demonstrate sustainable and scalable improvement of food systems through a container gardening project for iron-rich Hibiscus sabdarifa for consumption and income by empowering women during the dry/lean season in northern Ghana.

- **Aims 1:** To develop dietary screening tools that are validated by biomarkers for early detection of deficiencies among children 6-23 months and their mothers

    H1.1: *Dietary diversity score can predict iron deficiency among children 6-23 months and their mothers.*

    H1.2: *Dietary diversity score can predict iodine status deficiency among children 6-23 months and their mothers*.

- **Aims 2:** Indigenous nutrient-rich meals of hibiscus sabdarifa improves iron and iodine status of dyads

    H2.1: *Indigenous nutrient-rich meals of hibiscus sabdarifa improves iron status of dyads*

    H2.2: *Indigenous nutrient-rich meals of hibiscus sabdarifa improves iodine status of dyads*

- **Aims 3:** to demonstrate that container gardening can provide sustainable and scalable improvement of food systems for iron-rich Hibiscus sabdarifa for consumption and income during the dry/lean season in northern Ghana

  H3.1: *Container gardening can provide adequate amounts of vegetables for mother and child dyad during the dry season*

  H3.2: *Container gardening can provide adequate income to purchase iodized salt and Amani for mother and child dyad during the dry season*

1.3 Significance

This project addresses the gap in our knowledge and practices pertaining to serious and persisting trace mineral deficiencies that result in stunting and cognitive impairment in northern Ghana. Early detection of iron and iodine deficiencies with validated non-invasive dietary screening tools (aim 1), effective indigenous nutrient-rich meal-based programs (aim 2) and sustainable/scalable and women-led community-based food-system changing agricultural project (aim 3) are expected to be the most creative approach to counteract iron and iodine deficiencies in northern Ghana. This project will utilize science and education to change practices, environments and policies to reduce the prevalence of trace mineral deficiencies at the local, regional, national and global levels.

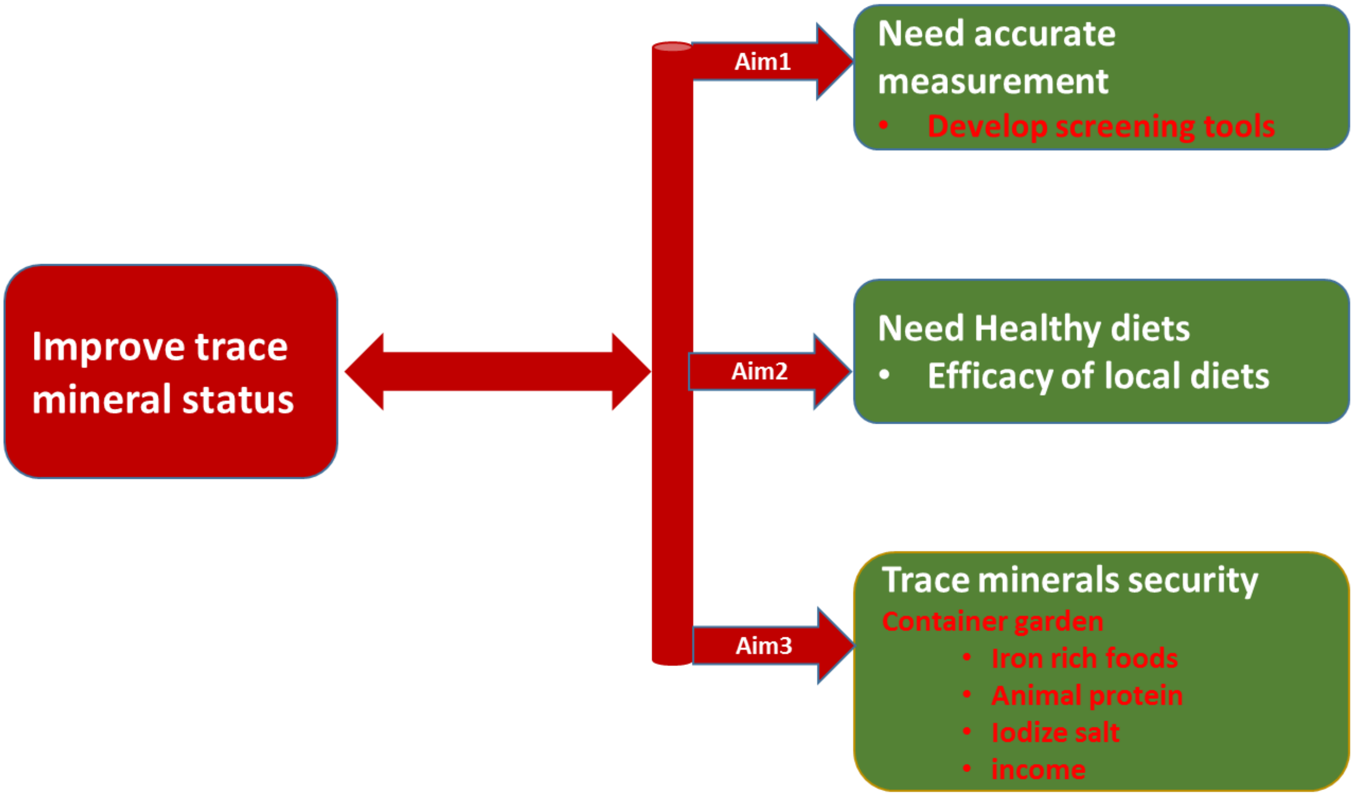

Figure 1: Research goal and aims

CHAPTER 2: LITERATURE REVIEW

2.1 Food security vs nutrition security

Food security has several definitions and key among them is FAO definition as "when all people, at all times, have physical, social and economic access to sufficient, safe and nutritious food that meets dietary needs and food preferences for an active and healthy life"(Qureshi et al., 2015), anything short of these results in food insecurity. Others simply put food insecurity as the uncertain availability of nutritionally adequate, safe foods for healthy life (Weiser et al., 2013).

Ghana has less than 9% of its population reported being food insecure in 2011-2013 (FAO, 2015). This is however based on caloric availability per person which does not reflect the true meaning of the FAO definition. On regional bases, the most food insecure households are found in northern Ghana: The highest proportion of food insecure households is in the Upper East Region (28%), Northern Region (10%) and 16% in the Upper West Region. The five districts with the highest proportion of severely and moderately food insecure households are Wa West (42%), Central Gonja (39%), Talensi-Nabdam (39%), Kassena-Nankana West (35%) and Kassena- Nankana East (33%) (WFP, 2012). Though the causes of these prevalence are complex, they are largely due to poverty, agricultural limitations, seasonal challenges and high food prices. In all these, small holder farmers, households headed by women, women and children suffer most. The consequences of food insecurity reflects in high malnutrition prevalence as evidence by the rates in northern Ghana (GSS, 2009; 2011; 2015).

With food insecurity prevalence of less than 9%, stable political environment, relative economic growth and improved health system over the years in Ghana (IFPRI,

2014), it is expected that malnutrition prevalence would have been better than observed. Food security is often use to imply nutrition security but they are not necessarily the same. Nutrition security requires simultaneously ‘food’, ‘health’ and ‘care’. So, there is no way to achieve nutrition security without food security (FAO, 2009). This implies that there is a need to measure the status of nutrients (other than calories).

In sum, food insecurity prevalence are high in northern Ghana and women and children are affected most. Although food security and nutrition security are related, food security measurements cannot be used to imply nutrition security. There is a need to develop screening tools to specifically measure nutritional status in Ghana (not caloric intake).

2.2 Micronutrients in human health

Micronutrients are basically essential vitamins and minerals required in small amounts by the body for proper growth and development. They include, but are not limited to: vitamins A, B, C and D, calcium, folate, iodine, iron, and zinc (Gernand et al., 2016). They are diverse with various vital biological roles, however, some are considered as problematic nutrients because of wide spread deficiencies. Key among these are iron, iodine and zinc (Gayer and Smith, 2015; Stewart et al., 2013). For this reason our focus would be on these trace minerals (iron, iodine, and zinc).

Trace elements or minerals are vital nutrients required in minute quantities for physiological functioning. These elements are ingested in milligrams or less per day. They are generally known to have a major function in many periods of women and

children's life and that multiple deficiencies, rather than single deficiencies, are common (Kontic-Vucinic et al., 2006). Iron, iodine, and zinc are the most widespread trace minerals deficiencies globally (Gayer and Smith, 2015). Controlling deficiencies in women of childbearing age breaks inter-generational malnutrition cycle (Berti et al., 2014; Micke et al., 2014; Radlowski and Johnson, 2013; Wang et al., 2015) since maternal nutrition has great influence on the birth outcomes of offsprings and their nutrition. It is known that maternal deficiencies in iron, iodine, and zinc among other factors affect intrauterine growth of fetus (Gayer and Smith, 2015; Stewart et al., 2013) and that infants with both symmetric and asymmetric intrauterine growth retardation demonstrate limited catch-up growth which persist into early childhood (Strauss and Dietz, 1997). Nutritional status in childhood affects nutritional and reproductive health in adulthood. This invariably affects subsequent generations. Studies among women of child bearing age is vital especially with respect to iron, iodine, zinc, and selenium (Malhotra et al., 2014; Radlowski and Johnson, 2013; Schmidt et al., 2014; Valera-Gran et al., 2014).

Iron. Iron is one of the problematic nutrients in the health of women and children. Iron in diets exists in two forms: ferrous form (Fe^2) which is from animal sources and often referred to as heme iron, and the ferric form (Fe^3) which is from plant sources also referred to as non heme iron. Total human body iron content is estimated to be 3.8 g in men and 2.3 g in women. Most iron in the body is in the form of heme iron. Heme iron is the essential constituent of hemoglobin in blood and myoglobin in muscle for oxygen transport, oxygen storage, electron transport for cytochrome function in aerobic respiration, and signal transduction as a cofactor for nitric oxide synthase and guanylyl

cyclase. Iron is essential for immune and cognitive development and functioning. The second largest pool of iron in the body is in its storage form ferritin, a ubiquitous intracellular protein (Ross et al., 2014).

Utilization of iron in food sources by humans and animals is highly influenced by bioavailability. The impact of iron is however influenced by other food substances such Oxalic acid, tannins, phytate, polyphenols, carbonate, phosphate, fiber and other metal ions being inhibitors of iron absorption. They inhibit absorption by forming non-absorbable complexes with the iron within the intestinal lumen. Vitamin C, fructose, citric acid, dietary protein, Lysine, histidine, cysteine, and methionine are enhancers of iron absorption (Haider et al., 2013). They enhance absorption by forming a chelate with ferric iron that remains soluble at the alkaline pH of the duodenum (Lynch and Cook, 1980)

Physiological mechanisms such as inflammation impacts negatively on iron status as well as its assimilation from the gastrointestinal tract. Infections (notably malaria, HIV disease, and tuberculosis), parasites (tape and hook worms), and inherited disorders of erythropoiesis (such as the thalassemic syndromes and hemoglobinopathies) affect ferritin status (Humphries et al., 2011; Lynch, 2011). Low serum ferritin value reflects depleted iron stores, but not necessarily the severity of the depletion as it progresses. This holds only in the absence of inflammation. In areas where inflammation is prevalent, soluble transferrin receptor is usually used as it does not rise in response to inflammation rise (World Health Organization, 2011). Additionally, in the presence of inflammations, hepcidin inhibits iron absorption which ultimately affects individuals' iron status (del Giudice et al., 2009).

Deficiencies of iron are associated with morbidity, mortality, preterm birth, low birthweight and inferior cognitive development (Haider et al., 2013). Iron deficiency is most common among women and children due to higher physiological needs and iron losses (menses in women). The burden is heaviest in developing countries largely due to poor diets and infections. Anemia is reported in 52% of pregnant women in non-industrialized and 23% of those in industrialized countries. Children in industrialized and non-industrialized countries are also reported to suffer from anemia (WHO, 2001) at 20% and 39%, respectively. In Ghana which is a non-industrialized country, iron deficiency anemia is seen in 78% of children under 5yrs and 59% of women of 15-49 years of age (GSS, 2009; GSS, 2011).

Iodine is another trace mineral which is essential to living organisms as it is an intramolecular component for the biosynthesis of thyroid hormones. The thyroid hormones control cell growth and differentiation, increase proteins, lipids, and carbohydrates metabolism (Haldimann et al., 2015). Iodine Deficiency Disorders (IDD), a collective number of health outcomes, is the world's leading cause of preventable cognitive retardation and poor psychomotor development in children. Additionally, iodine deficiency is commonly associated with goiter, intellectual impairments, growth retardation, neonatal hypothyroidism, and increased pregnancy loss and infant mortality (Haldimann et al., 2015; Pearce et al., 2013).

Globally, iodine deficiency is the world's leading cause of intellectual deficiency (Hollowell et al., 1998) and that 1.88 billion people have insufficient dietary iodine intakes with most of them being economically disadvantaged groups living in remote areas (Andersson et al., 2012) including Africa. In Ghana, only 35% of households use

adequately iodized salt. This percentage dwindles as you move across the country - from Accra (56%), Brong Ahafo (45%), Western (44%) through Northern (15%) to Volta (18%) and Upper East (18%) Regions of Ghana. Generally households in urban areas are more likely to use adequately iodized salt (45%), compared to households in rural areas (23%) (GSS, 2011).

Iodine needs are mainly met from diet or supplementation, deficiency can lead to iodine deficiency disorders (IDD). Dietary sources of iodine however varies from country to country. The major source of iodine is from sea foods, it is strongly enriched in near-coastal soils (Fuge and Johnson, 2015). Deficiencies are often low in countries with high consumption of sea products. In some instances, milk could be a rich source of iodine when cows are fed with iodine supplements. The alternative source of iodine is iodized salt (Lynch, 2011; Ross et al., 2014). Iodize salt is the commonest source of iodine for most African countries of which Ghana is of no exception. From the usage level of iodized salt, it could be inferred that 56% - 85% of households in southern and norther Ghana respectively could be at risk of iodine deficiency. This makes the study of iodine deficiency among the vulnerable groups very timely.

2.3 Dietary diversity score as a measure of diet quality

DDS is an indicator of food quality as well as a measure of food security and consumption patterns. It is defined as the number of foods or food groups consumed by an individual (IDDS) or by any member of the household inside the home (HDDS) over a reference time period - usually 24 hours (UN, 2008). Food grouping is dependent on the study objectives, thus whether emphasis is on energy-dense foods or micronutrient-rich foods. Usually, the number of food groups varies from 5 to 14, depending on the

main characteristic of the diet that the score intend to reflect -thus energy or micronutrient adequacy (UN, 2008).

In Nigeria and Kenya, positive association was found between dietary diversity and anthropometric status of children under five years (Onyango et al., 1998; Tarini et al., 1999). In a similar study a positive association was found between dietary diversity and micronutrients – Iron, zinc and vitamin A intakes (Tarini et al., 1999), in Ghana and Malawi however, weaker or no association was found for most nutrients (Ferguson et al., 1993).

A multi – country data analysis showed a consistent positive relationship between household level food group diversity and the consumption of energy (Hoddinott, 2002). In other validation studies done at the household level found the consumption of food items (12 foods identified as important) in the Venezuelan diet explaining 90.7% of the variance in total energy availability (Lorenzana and Sanjur, 1999), a similar outcome was found among Venezuelan population by other researchers (Lorenzana and Mercado, 2002). These studies are however context-specific, their applications should be carefully defined for each population based on their specific dietary patterns.

At the individual level, dietary diversity measures driven from 24-hour recalls or food frequency questionnaires showed positive correlation with greater energy intake by several studies. Torheim and colleagues tested two indicators: one based on the number of unique foods consumed by the individual (food variety score - FVS) and the other one based on the number of food groups consumed (dietary diversity score – DDS). They found both FVS and DDS to correlate positively with energy intake, with

coefficients of 0.38 and 0.29, respectively (Torheim et al., 2004). In Vietnam, it was found that women with high FVS and DDS had significantly higher mean energy intakes than those with lower scores (Ogle et al., 2001). It is suggested that greater dietary diversity is associated with greater energy intake. Dietary diversity indicators could be useful to assess the adequacy of energy intake at population levels.

There has been other research works that indicate that dietary diversity score is a good indicator for diet quality and micronutrients intake. **Table 1** gives detail summary of various studies in the developing world. Fundamental to all these researchers is that, none have predicted the biomarker status (which is the ultimate measure of nutrient status) of these nutrients using DDS, individual and household characteristics. They generally measure the quality of diet that is consumed. Additionally, researchers have used a variety of dietary diversity measures based on different food and food group classification systems, different numbers of foods and food groups and varying reference period lengths (Ruel, 2003b). Cutoff points of DDS to define varying levels of dietary quality have to be defined in the context where they are used, taking into account local food systems and dietary patterns (Ruel, 2003c). Using dietary diversity to predict diet quality and or disease outcome, it is important to use diverse classification of dietary diversity.

Table 1: Dietary diversity score as a measure of diet quality

Author	DDS description	Subject Description	Selected nutrients	Conclusion
Korkalo et al., 2016[1]	9 groups – 24hr recall<br>9 groups (15g minimum cut off) – 24hr recall<br>and 9 groups – 7 days recall	Cross sectional study of 227 adolescent girls (14-17yrs) from Mozambique	Iron, zinc, folate and Vitamin A	dietary diversity is associated with serum zinc, but this<br>association seems to be limited to the hunger season
Kennedy et al., 2007[2]	10 groups (not cut off) – 24hr recall<br>10 groups (1g & 10g cut off) – 24hr recall	Cross sectional study of 3164 children 24–71 months in Philippine	energy, protein, fat, calcium, iron, vitamin A, vitamin C, thiamin, riboflavin, and niacin, vitamin B-6, vitamin B-12, folate, and zinc	DDS 1g and DDS 10g were both significant predictors of adequate micronutrient intake
Moursi et al., 2008[3]	8 groups (not cut off) – 24hr recall<br>8 groups (1g & 10g cut off) – 24hr recall	Cross sectional study of 3164 children 6 -23 months in Madagascar	energy, protein, calcium, iron, vitamin A, vitamin C, thiamin, riboflavin, vitamin B-6, vitamin B-12, and zinc	DDS are useful proxies of micronutrient density of foods consumed by BF and non-BF infants
Arimond et al., 2010[4]	6, 9, 13, or 21 groups (1g & 15g cut off) – 24hr recall	Cross sectional study of 2078, 2024, 2086, 2083, 1211 women from Burkina Faso, Mali, Mozambique, Bangladesh, and the Philippines respectively	Vitamin A, thiamin, riboflavin, niacin, vitamin Br6, folate, vitamin B-12, vitamin C, calcium, iron, and zinc.	Simple food group diversity indicators hold promise as proxy indicators of micronutrient adequacy
Steyn et al., 2006[5]	9 groups (no cut off) – 24hr recall<br>45 food items (no cut off) – 24hr recall	Cross sectional study of 2200 children (1-8yrs) from South Africa	vitamins A, B-6, B-12 and C,<br>niacin, thiamin, riboflavin, folate, calcium, iron and zinc	FVS or DDS can be used as a simple and quick indicator of the micronutrient adequacy of the diet.
Mirmiran et al., 2004[6]	23 groups (1/2 serving cut off) – 24hr recall	Cross sectional study of 304 adolescents (10-18yrs) from Tehran	vitamin A, riboflavin, thiamin, vitamin C, calcium, iron, zinc, phosphorus, magnesium, protein, potassium, fat, carbohydrate	DDS is an appropriate method to evaluate nutrient intake adequacy in this group of adolescents

Bukania et al., 2014[7]	9 groups (no cut off) – 24hr recall	Cross sectional study of 277 women (15-49yrs) and children (6-36 mn) pair from Kenya	Macronutrients - Protein and carbohydrates	Dietary diversity is highly correlated with caloric and protein adequacy. Differences in agroecological zones may not affect dietary diversity and nutritional status of farmer households. Consequently use of DDS may lead to underestimation of food insecurity in semiarid settings.

Title of papers

[1] Associations of dietary diversity scores and micronutrient status in adolescent Mozambican girls

[2] Dietary Diversity Score Is a Useful Indicator of Micronutrient Intake in Non-Breast-Feeding Filipino Children

[3] Dietary Diversity Is a Good Predictor of the Micronutrient Density of the Diet of 6- to 23-Month-Old Children in Madagascar

[4] Simple Food Group Diversity Indicators Predict Micronutrient Adequacy of Women's Diets in 5 Diverse, Resource-Poor Settings

[5] Food variety and dietary diversity scores in children: are they good indicators of dietary adequacy?

[6] Dietary diversity score in adolescents - a good indicator of the nutritional adequacy of diets: Tehran lipid and glucose study

[7] Food Insecurity and Not Dietary Diversity Is a Predictor of Nutrition Status in Children within Semiarid Agro-Ecological Zones in Eastern Kenya

2.4 Interventions to improve trace mineral status with dietary approaches

Globally, trace minerals deficiencies are of great concern with the most widespread deficiencies being iron, zinc, and iodine (Gayer and Smith, 2015). Several approaches have been adopted to counteract these deficiencies including food fortification, supplementation and dietary diversity approaches. The key challenges with the former two are limited in sustainability in terms of sociocultural acceptability, affordability and availability within natural ecosystem. Dietary approaches such as food-to-food fortification (complementarianism) can be promising to improve trace minerals intake (Cercamondi et al., 2014) especially in developing countries. The fundamental challenge in here is the efficacy of these food-to-fortification to improve trace minerals status. Little data exist for most indigenous foods in sub – Saharan Africa of which Ghana is of no exception.

Below gives detail summary of various studies using dietary approaches to improve micronutrient status. What is missing in these studies is that they all rely heavily on fortification or supplementation. Food-to-food fortification or food complementarianism would be ideal in real world situations as individuals' natural consumption is not supplements or fortificants.

2.5 Dietary diversity score as a measure of disease outcome

Dietary diversity (DDS) reflects micronutrient adequacy of the diet and is associated with better child growth as well as health outcome (Amugsi et al., 2014; Nguyen et al., 2013). Nguyen used 7 food groups from a 24 hr recall while Amugsi et al used 16 food groups. A study in Cambodia indicates that consumption of a diverse diet (FVS, 9 food items –DHS dataset) among children (12 – 59 mo) was associated with a reduction in stunting (Darapheak et al., 2013). Similarly, in Ghana, DDS (16 food groups, 24hr recall, DHS) was found to be associated with stunting among rural children, but not urban children, after controlling for child, maternal, and household characteristics(Amugsi et al., 2014). Caloric intake was not catered for as food intake (24hr recall) was mainly qualitative by nature.

Additionally, a study in Kenya among HIV patients shows an association between DDS (9 food groups, 24hr recall) and health-related quality of life (mental, physical and general health scores) and that it may be considered as part of comprehensive interventions designed to mitigate psychosocial consequences of HIV (Palermo et al., 2013). What appears missing is the failure to include one's immediate environment into predicting disease outcome with dietary diversity. DDS is strongly linked to one's immediate environment thus one's household characteristics.

2.6 Interventions to improve trace mineral status with dietary approaches

Globally, trace minerals deficiencies are of great concern with the most widespread deficiencies being iron, zinc, and iodine (Gayer and Smith, 2015). Several approaches have been adopted to counteract these deficiencies including food fortification, supplementation and dietary diversity approaches. The key challenges with

the former two is sustainability. Dietary approaches such as food-to-food fortification can be a promising approach to improve trace minerals intake especially in low dietary trace minerals intake and bioavailability from monotonous diets based on a small number of staple plant foods (Cercamondi et al., 2014) as in the case of most developing countries. The fundamental challenge in here is the efficacy of these food-to-fortification to improve trace minerals status. Little data exist for most indigenous foods in sub – Saharan Africa of which Ghana is of no exception.

For lasting solutions to trace minerals deficiencies in developing countries, there is a need to examine the efficacy of indigenous meals to improving iron and iodine status of women and children. Below (Table 2) gives detail summary of various studies using dietary approaches to improve micronutrient status. What is missing in these studies is that they all rely heavily on fortification or supplementation. Food-to-food fortification or food complementarianism would be ideal in real world situations as individuals' natural consumption is not supplements or fortificants.

Table 2: Dietary base interventions to improve nutrients intake

Author	Title	Subject Description	Selected nutrients	Conclusion
Egbi et al., 2015[1]	3groups fed with 3 meals:<br>Group1: cowpea-based food + 3% fish powder + 33 mg vitamin C/100 mL<br>Group2: cowpea-based food + 33 mg vitamin C/100 mL<br>Group3: cowpea-based food + placebo	6-month nutrition intervention study among 162 children (6-12yrs)	Iron	Cowpea- based food containing 3% fish meal and served with vitamin C–rich drink improved hemoglobin concentration and minimized the prevalence of anemia among the study participants
Tetens et al., 2007[2]	Meat and vegetables given to 2 groups (meat group & vegetable group) to take home (biweekly). Amount of iron was similar in products for each group. Compliance measured by quantity of foods returned.	A stratified random treatment of 57 women (19-39 yrs) for 10-20 wks	Iron	Serum ferritin remained unchanged in women on the meat-based diet but decline for women on the vegetable-based diet.<br>it is thus important to ensure a balance between dietary iron content and iron bioavailability for the maintenance of blood indicators of iron stores in women with initially low iron status
Parker et al., 2015[3]	2groups given dry rations:<br>Group1: Fortified rice (iron, zinc, thiamine & folic acid)<br>Group2: Rice + Beans (Vit A & iodized salt)	Cluster-Randomized feeding trial in 994 children for 7 months	Iron	There was no significant difference in Hb concentration between the intervention and control groups
Cercamondi et al., 2014[4]	3 test meals<br>1. Maize-based t ô + amaranth sauce,<br>2. Maize-based t ô + iron-improved amaranth sauce (increased quantities)<br>3. Maize-based t ô + iron-improved jute sauce (increased quantities)	A randomized crossover design with multiple meals was used with each woman serving as her own control<br>(no animal source)	Iron	A food-to-food fortification approach based on an increase in leafy vegetables quantity does not provide additional bioavailable iron, presumably due to the high phenolic compound concentration of the leaves tested
Christofides et al., 2006[5]	Multi-micronutrient Sprinkles treatment for 3 groups (Nutrients: Fe, Zn, folic acid, Vit A & D)<br>Group1: 12.5 mg of iron<br>Group2: 20mg mg of iron<br>Group2: 30mg mg of iron	A randomized clinical trial for 8 weeks	Iron	Low doses of sprinkles (12.5 mg) may be effective in in reducing anemia children
Abizari et al., 2012[6]	2groups fed with 2 meals:<br>Group1: Fortified cowpea-based food<br>Group2: Unfortified cowpea-based food	Randomized, double-blind, controlled trial. 241 children (2 -12	Iron	consumption of fortified cowpea meal significantly improved both functional and storage iron status

		years) feed with tubani (3d/wk)		
Zhao et al., 1999[7]	3groups, salt given at household level:<br>Group1: 25 ppm iodize salt every 2 months<br>Group2: purchased iodize salt every 2 months<br>Group3: 4 capsules of iodized oil (100 mg iodine each) + purchased iodize salt every 2 months	Randomized clinical trial involving 145 children (8 – 10 yrs) for 6,9,12,& 18 months	Iodine	The median urinary Iodine improved at the 6-month follow-up. Children with initially a low to moderate level of iodine deficiency were not iodine deficient on all indicators after 18 months of study.

Titles of papers

[1] Impact of Cowpea-Based Food Containing Fish Meal Served With Vitamin C–Rich Drink on Iron Stores and Hemoglobin Concentrations in Ghanaian Schoolchildren in a Malaria Endemic Area

[2] The impact of a meat- versus a vegetable-based diet on iron status in women of childbearing age with small iron stores

[3] A Blinded, Cluster-Randomized, Placebo-Controlled School FeedingTrial in Burundi Using Rice Fortified With Iron, Zinc,Thiamine, and Folic Acid

[4] A Higher Proportion of Iron-Rich Leafy Vegetables in a Typical Burkinabe Maize Meal Does Not Increase the Amount of Iron Absorbed in Young Women

[5] Multi-micronutrient Sprinkles including a low dose of ironprovided as microencapsulated ferrous fumarate improves haematologic indices in anaemic children: a randomized clinical trial.

[6] Whole Cowpea Meal Fortified with NaFeEDTA Reduces Iron Deficiency among Ghanaian School Children in a Malaria Endemic Area

[7] Randomized clinical trial comparing different iodine interventions in school children

2.7 Household food production to improve nutritional status

Agricultural production influences the quality of diets of smallholder farming households through production of subsistence food crops and animals that the households consume directly or through the sale of agricultural goods that affect household incomes which affects food purchases and consumption (Jones et al., 2014). In addition, pathways other than the agriculture to nutrition affect the nutritional status of individuals within households through (1) household income may be spent to purchase healthcare for individuals, (2) a woman's time and workload may affect her energy expenditure and health as well as her capacity to feed and care for young children, and (3) a woman's control of household income, affected in part by her ownership of farm output and the kind of income generated from that output, affects the kinds of purchases made with the income and the allocation of resources within households (Jones et al., 2014).

The fundamental model of household agriculture production in improving family nutritional status stems from the basis that agriculture production leads to increased availability of nutritious foods which improves household's consumption patterns. These consumption patterns then lead to improved health and nutritional status which in turn improve family wellbeing and the agricultural production. This cycle is based on the condition that the consumed food is of quality (diverse diet) and the body is able to utilize the foods. It is on this basis that a proposition is made to explore alternative nontraditional routes to producing nutrient rich vegetables during the dry season in Ghana.

CHAPTER 3: METHODS AND APPROACHES

This study will be a community based trial in the Upper East Region of Ghana to identify non-invasive, dietary approaches validated by biomarker status of iron and iodine and to demonstrate alternative approaches to providing access to foods rich in trace minerals (iron and iodine) at the household level. This project is to address the high prevalence of stunting in Upper East Region. Our preliminary data indicate that common meals in the region are consisted mainly of cereals (maize, millet, rice and sorghum), vegetables, legumes/pulse. Consumption of fish, meat and animal products is occasional (Appendix E). These findings are in agreement with an earlier study on Food Security & Vulnerability Analysis in northern Ghana (WFP, 2012). These consumption practices are predominant in rural part of northern Ghana. The vegetables which the rural people rely on are seasonally cultivated and run out of stock during the prolonged 6-7 months dry season, when food insecurity is at the peak. For these reasons, the dry season is referred to as “lean or hunger” period.

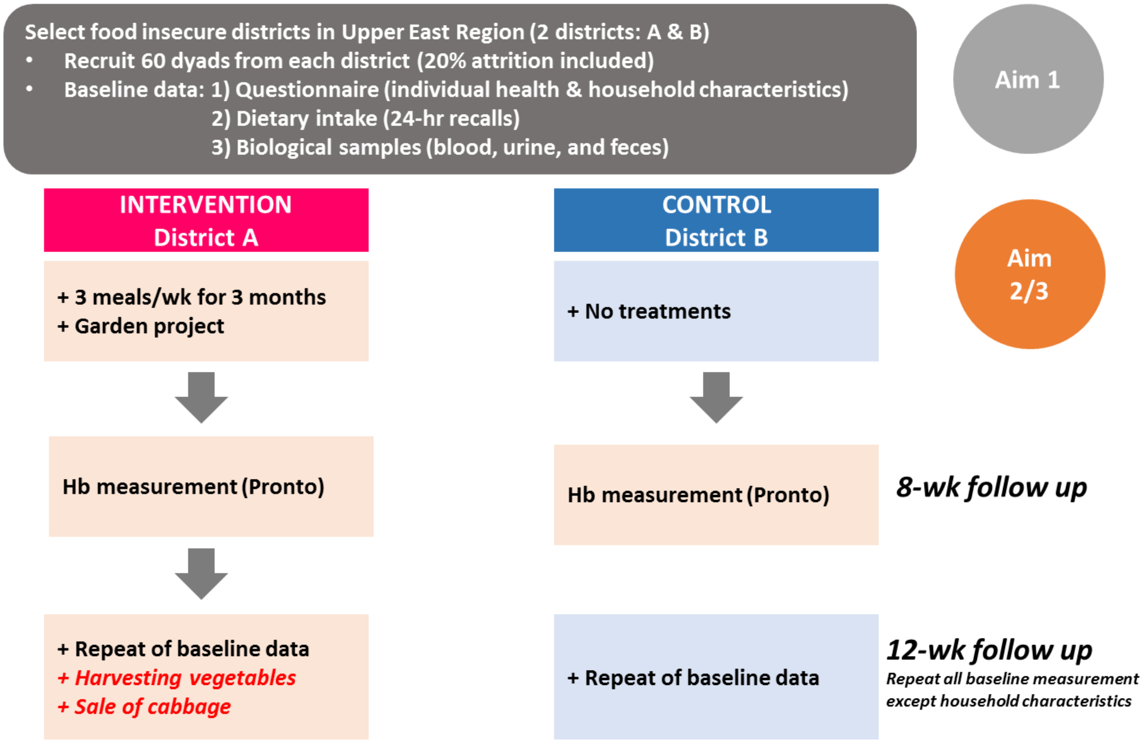

Figure 2: Study frame

3.1 Aim1 - Screening tool development

Iron and iodine status among children 6-23 months and their mothers in Ghana can be predicted by dietary and household characteristics

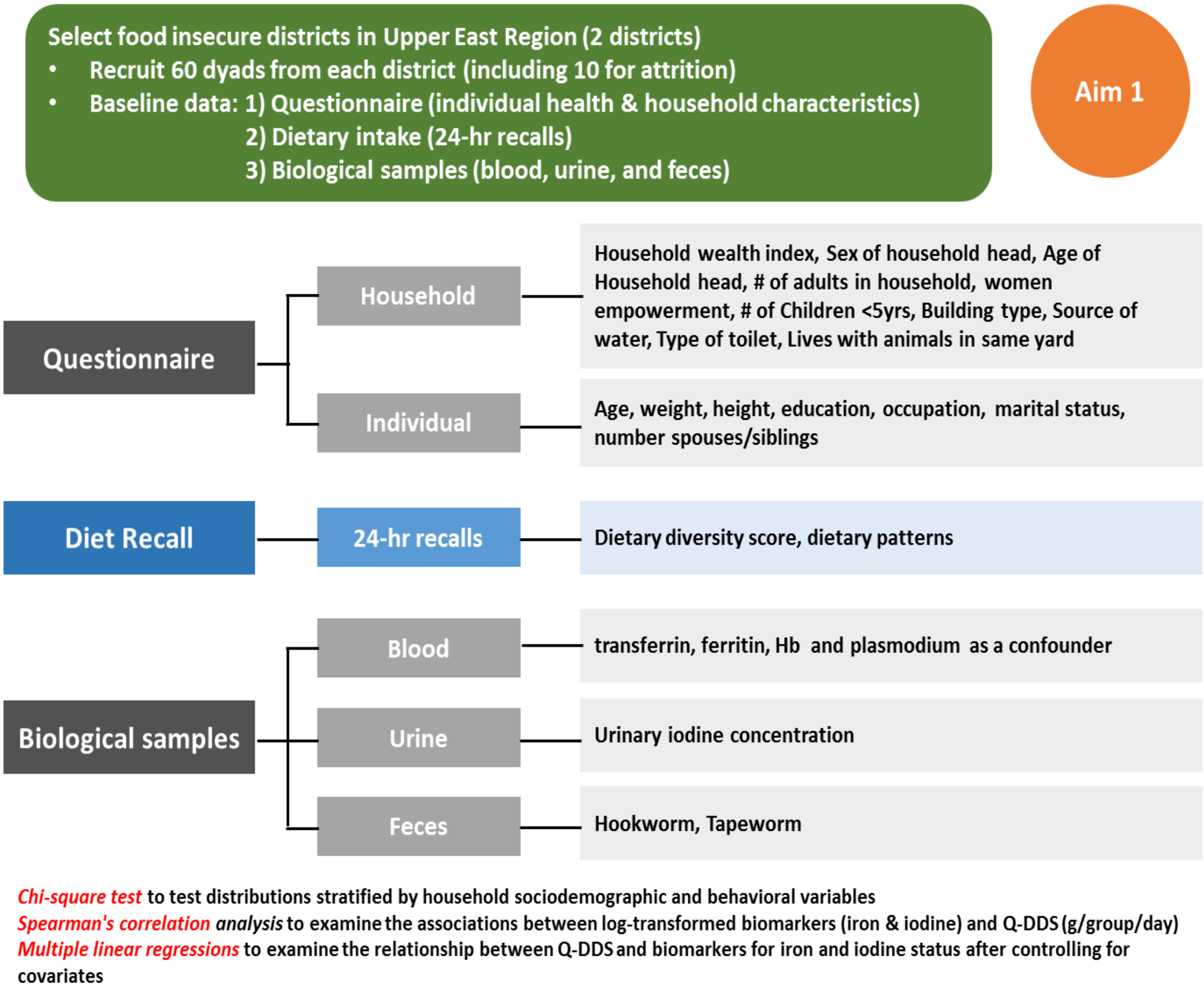

Figure 3: Aim 1 and its research approaches

The study subjects, women 15-49 y and their children 6 – 23 months, will be recruited from one of two selected districts in the Upper East Region of Ghana during their dry season. Kassena Nankana West and Builsa North Districts of the Upper East Region of Ghana have been selected for this study to be conducted for the period of

May 2016 – September 2017**.** For inclusion, districts needed to be among the top food insecure districts. For inclusion of communities at each district, households is such communities should have access to water throughout the dry season without shortages. Sampling will start from Sakaa and Chania for Kassena Nankana West district and Chuchuliga for the Builsa North district. The choice of these communities is based on preliminary inquiries which indicate that those communities have water supply throughout the dry season, have sizeable number of children (6 – 23 months old) which gives us a good sampling frame, in addition, researchers have previous experience with those communities and that will facilitate the community entry process.

Dyads would be drawn from selected districts using community based birth registers kept by *community based health volunteers*. These volunteers live in their respective communities, register all births and deaths in their communities, and support all health related activities targeting their respective communities. Recruitment of dyads (with children 6-23 months) for this study will be identified and located with the help of *community based health volunteers* for recruitment. Recruitment will begin with one community in each district and then to neighboring communities until the desired sample size is achieved per district. Participants should be members of existing community based women group or willing to join one in their respective communities. Only dyads who report the absence of illness (malaria, diarrhea, fever, worm infections etc.) and consent to participate in the study by signing or thumb printing participant consent form. Thumb printing is widely used in Ghana because of high rate (75%) of illiteracy especially in rural northern Ghana (GSS, 2014). All analytical work for

biomarkers will be carried out at the Noguchi Food Research Institute in Ghana / Tamale Teaching Hospital / Lancet diagnostic laboratory.

The research will be an intervention trial. A total of 120 dyads (Intervention - 60 mother/child dyads; control - 60 mother/child dyads) will be drawn from Upper East region (two districts). Optimal design software was used to calculate the minimal sample size of 100 dyads (intervention – 50 dyads, control – 50 dyads) with a power of 80%, significance level of 5%, and coefficient of determination of 65%, and minimum detectable effect of 0.33 of diet on hemoglobin change. 20% of the required sample size was added to cater for dropouts.

Data collection: At baseline, 24 hr recall and household questionnaire would be used to collect individual characteristics *(age, weight, height, education, occupation, marital status, number spouses/siblings, smoking habits, alcohol and tobacco usage)*; household characteristics *(household wealth index, Sex of household head, age of household head, number of adults in household, women empowerment, number of <children 5yrs, building type, source of water, type of toilet, lives with animals in same yard)*. Blood would be drawn to test for ferritin, hemoglobin and transferrin levels. Presence of malaria would be tested for as a confounder to iron status. Though inflammatory markers are vital confounding factors for iron, they would not be assessed for logistics constraints. Pronto and HemoCue would be used to assess hemoglobin levels, ferritin and transferrin would be analyzed using ELISA. Stool samples would also be tested for parasites (tape and hookworms) as confounders to iron status. Follow up data would be collected at 8 weeks (only hemoglobin) and 12 weeks (same as baseline excluding household characteristics). Data would be collected in both districts.

Computation of four Dietary Diversity Scores, will be used: qualitative DDS scores based on *aggregated food groups* (DDS6 and DDS15)*; aggregated subgroups based on iron and iodine rich foods; 24 hr vs weekly consumption; and semi-quantitative DDS that have minimal consumption cut offs (1g and 10g)*. In each of the categories, a score of 1 will be assigned for consumption of a food group and a score of 0 for non-consumption. Consumption will only be counted for a food group or item when the amount is equal to or more than the minimal imposed amounts under the DDS category with *minimal consumption*. Weekly *consumption* of a food group is when such a food group is consumed 3 or more days in a week. Similarly, a score of 1 would be assigned for weekly (and 24hr recall) and a score of 0 if otherwise.

Statistical analysis: Developing dietary assessment tools validated by biomarkers. Quantitative dietary diversity scores in four classifications of food groups' intake and dietary patterns will be investigated initially. The following hypothesis would be investigated using the under listed statistical approaches.

H1.1: *Dietary diversity score can predict iron deficiency among children 6-23 months and their mothers.*

H1.2: *Dietary diversity score can predict iodine status deficiency among children 6-23 months and their mothers*.

Validation of dietary screening tools will involve 1) chi-square test to test distributions stratified by household sociodemographic and behavioral variables, 2) Spearman's correlation analysis to examine the associations between log-transformed biomarkers (iron & iodine) and quantitative dietary diversity score (Q-DDS) (g/group/day), and 3) multiple linear regressions to examine the relationship between Q-DDS (independent

variable) and biomarkers for iron and iodine status (dependent variables) after controlling for covariates such as survey month, sex, age, household wealth index, sex of household head, number of siblings (<5yrs) and salt use at household.

3.2 Aim 2 – Feeding trial

Indigenous nutrient-rich meals of hibiscus sabdarifa improves iron and iodine status of dyads

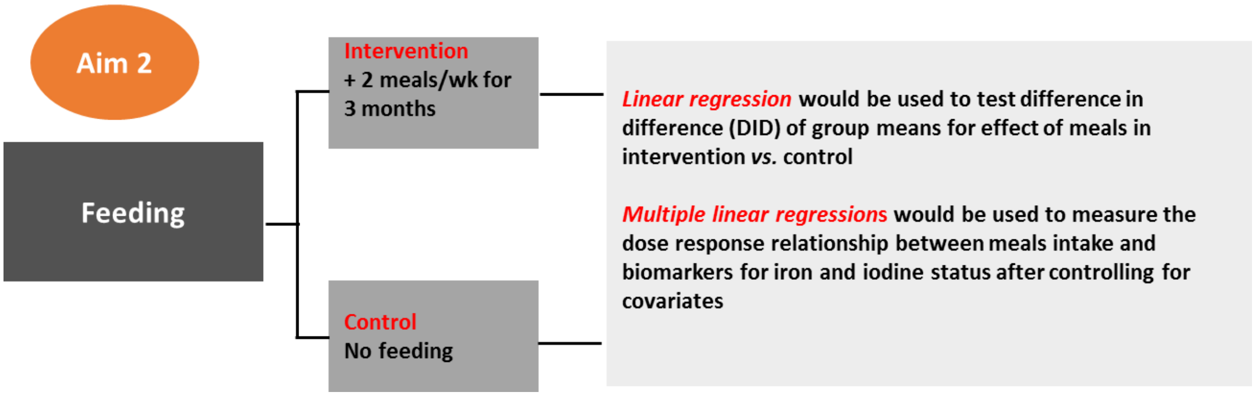

Figure 4: Aim 2 and its research approaches

After baseline data collection, one district would be assigned as a treatment group (intervention) and the other as control. Participating dyads in the intervention group would be provided with 3 meals/week (containing 46.35 mg of iron/meal for adults) for a period of 3 - 6 months under strict supervision.

Mass deworming, malaria screening and treatment would be carried out at baseline. A typical serving (meal) for adults will contain 25.95 mg of iron from *hibiscus sabdarifa, 12.45* mg of iron from fish, 7.05 mg of iron from dawadawa, and 0.9 mg of iron from groundnut; children's meals will contain 8.65 mg of iron from *hibiscus sabdarifa, 4.15* mg of iron from fish, 2.35 mg of iron from dawadawa, and 0.3 mg of iron from

groundnut. Preliminary run on consumption showed that a minimum of 1 and 3 servings/person were acceptable portions for kids and adults respectively. Vegetable/fish combination indicate that: a ratio of 10g vegetables to 3g of fish was the maximum amounts that could be combined to give an acceptable taste of vegetable soup among participants. Iodized salt would be used in all served meals. Iodize salt would also be provided in glass containers with screw tops for participants to use at household level. The salt would be replenish as household salt runs down.

Nature of meals: The soup from the vegetables is mainly made up of: *hibiscus sabdarifa, groundnut,* water, dawadawa (condiment), salt and pepper. It is either eaten alone or with *Tou Zaafi* (Paste made of millet/maize flour). The proposition here is to add fish to the soup and examine its effect on iron status among dyads. Meals would be prepared by the women themselves to build trust and avoid any form of suspicion. They would either prepare meals in rotation by various days to give all the women the chance to be part of the preparation. They would however be a chef to monitor the compliance of all measurements pertaining to the recipe. While the meal process is ongoing, the rest of the women would be engage in the garden activities by an experience Gardner. Approximately, each day’s meeting session would be about 2 hours. In addition to data collection at baseline, 8 and 12 weeks, there would be a weekly interview ***on consumption alterations*** at household level due to *treatment meals* in the intervention group.

Statistical analysis: The efficacy of these meals to enhancing iron and iodide status will be determined by dose responses to the meal consumption by mother-child

dyads over the study period. The hypotheses below would be investigated using the under listed statistical approaches.

H2.1: *Indigenous nutrient-rich meals of hibiscus sabdarifa improves iron status of dyads*

H2.2: *Indigenous nutrient-rich meals of hibiscus sabdarifa improves iodine status of dyads*

1) Paired t-tests would be used to compare changes in iron and iodine status between the control and intervention groups (i.e., meals with Hibiscus sabdarifa, dried fish and iodize salt) and overtime (baseline vs. week 6 and 12), and 2) predictability, sensitivity and specificity to valuate and compare the utility of each tool developed. Statistical significance will be defined as $p < 0.05$.

3.3 Aim 3 – Container gardening

Container gardening provides sustainable and scalable food systems for iron-rich hibiscus sabdarifa for consumption and income at household level

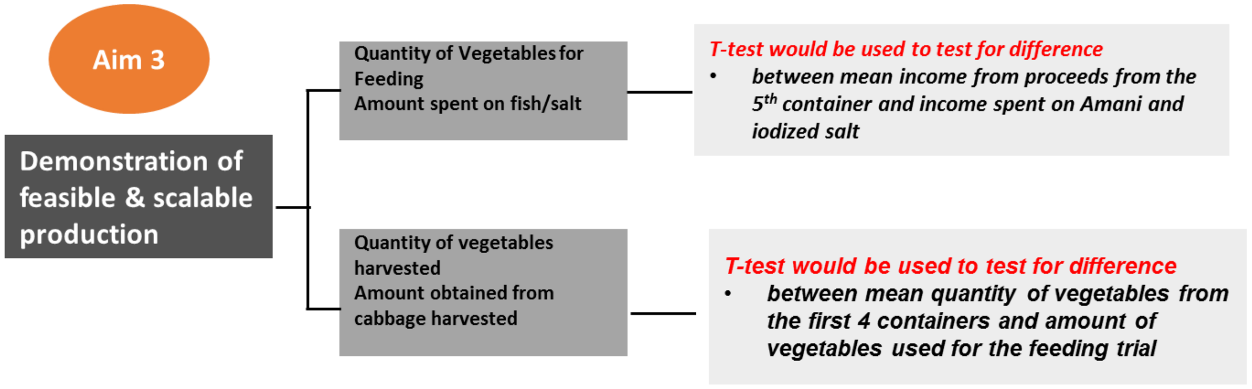

Figure 5: Aim 1 and its research approaches

Dyads in the intervention group will be provided with five containers each to plant vegetables during the lean season in a demonstration project. First four containers would be used for planting vegetables (Hibiscus sabdarifa) known to households in participating districts. Production from the fifth container would be reserved for sale in the market, to generate income over the growing season to cover buying food (ingredients, high quality protein and iodize salt) during the dry season. The fifth container would be used for vegetables with ready demand (cabbage and carrots) in the food service sector. Women would have their containers placed and fenced within the clinic settings and a large container mounted to supply water for gardening. Containers would be filled with sand at the first week of recruitment with the support and coaching from an experience Gardner. The gardening activities, including women coaching sessions, would be under the care of the Gardener.

The demonstration project would invest in two motorcycles – *"motor king"* (to ensure at least one is always running). A young man would gather the food produce (cabbage and carrots) from the women and use the motorcycle to transport the produce and a representative of the women to the market. In addition to helping organize the marketing, the tactic also provides much-needed employment to young men who would otherwise be idle.

Marketing: When women market their produce as individuals, the competition drives down the price. Purchasers, including institutional buyers for schools and hospitals, exploit the vulnerability, waiting until late in the day to issue a take-it-or-leave offer that the women are forced to take. By pooling their production into a marketing cooperative where there is only one person for buyers to bargain with, the goal is to

raise the income for all. The produce would be sold directly to restaurants which the women would have established a relationship with and have recruited such restaurants as clients. These restaurants use cabbage on daily bases as raw materials for their foods. When produce are ready for harvesting, recruited clients would be contacted for their orders before cabbage is harvested from containers. Facilitating and coaching of women on recruiting clients would be handled by trained marketers in the second week (as research team is engaged in data collection at the control group).

Statistical analysis: Demonstrating that container gardening is capable of providing sustainable and scalable community based agriculture/food system during the long dry season in northern Ghana, the under listed hypothesis will be tested.

H3.1: *Container gardening can provide adequate amounts of vegetables for mother and child dyad during the dry season*

H3.2: *Container gardening can provide adequate income to purchase iodized salt and Amani for mother and child dyad during the dry season*

T-test would be used to test for difference:

1) between mean income from proceeds from the 5th container and income spent on amani and iodized salt
2) between mean quantity of vegetables from the first 4 containers and amount of vegetables used for the feeding trial

3.4 Study ethics

Ethical clearance has been obtained from the Navrongo Health Research Centre (NHRC) Institutional Review Board (IRB) and Michigan State University's Institutional

Review Board. Consent for participation would be sought from all participants. Parental consent would be sought for the participation of children. Literate parents would be given the consent form to read, which would be followed by further explanations of the study by researchers to ensure a common understanding. On approval, either parent would sign the consent form for their child. Mothers would sign for themselves. To prevent any unforeseen cultural hurdles, verbal consent would also be sought from male partners for the participation of their spouses and children. For parents who cannot read, researchers would explain the study to them and mothers would thumb print the consent form for themselves and children. Illiterate mothers would thumb print for themselves and verbal consent sought from their male partners. We do anticipate cultural barriers as well as fear of stigmatization of some diseases in our study area. We will overcome this barrier with proper community entry techniques couple with sensitization and education.

REFERENCES

Allen LH, De Benoist B, Dary O and Hurrell R (2006) Guidelines on food fortification with micronutrients.3-20.

Caulfield LE, Richard SA, Rivera JA, Musgrove P and Black RE (2006) Stunting, wasting, and micronutrient deficiency disorders.

David P (2003) Evaluating the Vitamin A Supplementation Programme in northern Ghana: Has it contributed to improved child survival?, Micronutrient Initiative and JSI.

FAO (2015) Food security indicators - by Food and Agriculture Organisation, Food and Agriculture Organisation.

GSS (2009) Ghana Demographic and Health Survey 2008., pp 1-483, Ghana Statistical Service - GSS, Ghana Health Service - GHS, and ICF Macro.

GSS (2011) Ghana Multiple Indicator Cluster Survey with an Enhanced Malaria Module and Biomarker Final Report: Monitoring the situation of children and women, pp 52-60, Ghana Statistical Service.

GSS (2014) Ghana living standards survey round 6 (GLSS 6) - main report, pp 16-20, Ghana Statistical Service.

Hoffmann K, Schulze MB, Schienkiewitz A, Nöthlings U and Boeing H (2004) Application of a new statistical method to derive dietary patterns in nutritional epidemiology. *American Journal of Epidemiology* **159**:935-944.

IFPRI (2014) Global Nutriton Report: Ghana 2014 Nutrition Country Profile pp 1-2, International Food Policy Research Institute.

Kennedy G, Nantel G and Shetty P (2003) The scourge of" hidden hunger": global dimensions of micronutrient deficiencies. *Food Nutrition and Agriculture*:8-16.

Nyumuah RO, Hoang TC, Amoaful EF, Agble R, Meyer M, Wirth JP, Locatelli-Rossi L and Panagides D (2012) Implementing large-scale food fortification in Ghana: lessons learned. *Food Nutr Bull* **33**:S293-300.

Ruel MT (2003) Is dietary diversity an indicator of food security or dietary quality? A review of measurement issues and research needs. *Food Nutr Bull* **24**:231-232.

Saaka M (2012) Combined iron and zinc supplementation improves haematologic status of pregnant women in upper west region of Ghana. *Ghana medical journal* **46**:225.

Stewart CP, Iannotti L, Dewey KG, Michaelsen KF and Onyango AW (2013) Contextualising complementary feeding in a broader framework for stunting prevention. *Maternal and Child Nutrition* **9**:27-45.

Strauss RS and Dietz WH (1997) Effects of intrauterine growth retardation in premature infants on early childhood growth. *The Journal of Pediatrics* **130**:95-102.

World Health Organization (2010) WHO guidelines on drawing blood: best practices in phlebotomy.

Abizari A-R, Moretti D, Zimmermann MB, Armar-Klemesu M and Brouwer ID (2012) Whole cowpea meal fortified with NaFeEDTA reduces iron deficiency among Ghanaian school children in a malaria endemic area. *The Journal of Nutrition* **142**:1836-1842.

Allen LH, De Benoist B, Dary O and Hurrell R (2006) Guidelines on food fortification with micronutrients.3-20.

Amugsi DA, Mittelmark MB and Lartey A (2014) Dietary diversity is a predictor of acute malnutrition in rural but not in urban settings: evidence from Ghana. *British Journal of Medicine and Medical Research* **4**:4310.

Andersson M, Karumbunathan V and Zimmermann MB (2012) Global Iodine Status in 2011 and Trends over the Past Decade. *The Journal of Nutrition* **142**:744-750.

Arimond M, Wiesmann D, Becquey E, Carriquiry A, Daniels MC, Deitchler M, Fanou-Fogny N, Joseph ML, Kennedy G and Martin-Prevel Y (2010) Simple food group diversity indicators predict micronutrient adequacy of women's diets in 5 diverse, resource-poor settings. *The Journal of Nutrition* **140**:2059S-2069S.

Bath SC, Steer CD, Golding J, Emmett P and Rayman MP (2013) Effect of inadequate iodine status in UK pregnant women on cognitive outcomes in their children: results from the Avon Longitudinal Study of Parents and Children (ALSPAC). *The Lancet* **382**:331-337.

Berti C, Cetin I, Agostoni C, Desoye G, Devlieger R, Emmett PM, Ensenauer R, Hauner H, Herrera E, Hoesli I, Krauss-Etschmann S, Olsen SF, Schaefer-Graf U, Schiessl B, Symonds ME and Koletzko B (2014) Pregnancy and infants' outcome: nutritional and metabolic implications. *Crit Rev Food Sci Nutr*:1-38.

Bukania ZN, Mwangi M, Karanja RM, Mutisya R, Kombe Y, Kaduka LU and Johns T (2014) Food insecurity and not dietary diversity is a predictor of nutrition status in children within semiarid Agro-Ecological zones in Eastern Kenya. *Journal of nutrition and metabolism* **2014**.

Caulfield LE, Richard SA, Rivera JA, Musgrove P and Black RE (2006) Stunting, wasting, and micronutrient deficiency disorders.

Cercamondi CI, Icard-Vernière C, Egli IM, Vernay M, Hama F, Brouwer ID, Zeder C, Berger J, Hurrell RF and Mouquet-Rivier C (2014) A higher proportion of iron-rich leafy vegetables in a typical Burkinabe maize meal does not increase the amount of iron absorbed in young women. *The Journal of Nutrition* **144**:1394-1400.

Christofides A, Asante KP, Schauer C, Sharieff W, Owusu-Agyei S and Zlotkin S (2006) Multi-micronutrient Sprinkles including a low dose of iron provided as microencapsulated ferrous fumarate improves haematologic indices in anaemic children: a randomized clinical trial. *Maternal & child nutrition* **2**:169-180.

Darapheak C, Takano T, Kizuki M, Nakamura K and Seino K (2013) Consumption of animal source foods and dietary diversity reduce stunting in children in Cambodia. *International archives of medicine* **6**:1.

del Giudice EM, Santoro N, Amato A, Brienza C, Calabro P, Wiegerinck ET, Cirillo G, Tartaglione N, Grandone A and Swinkels DW (2009) Hepcidin in obese children as a potential mediator of the association between obesity and iron deficiency. *The Journal of Clinical Endocrinology & Metabolism* **94**:5102-5107.

Egbi G, Ayi I, Saalia FK, Zotor F, Adom T, Harrison E, Ahorlu CK and Steiner-Asiedu M (2015) Impact of Cowpea-Based Food Containing Fish Meal Served With Vitamin C–Rich Drink on Iron Stores and Hemoglobin Concentrations in Ghanaian Schoolchildren in a Malaria Endemic Area. *Food and nutrition bulletin* **36**:264-275.

FAO (2009) SUMMARY OF THE FSN FORUM DISCUSSION No.34

FOOD SECURITY AND NUTRITION SECURITY – WHAT IS THE PROBLEM AND WHAT IS THE DIFFERENCE

FROM 1 APRIL TO 15 MAY 2009.

Ferguson EL, Gibson RS, Opare-Obisaw C, Osei-Opare F, Lamba C and Ounpuu S (1993) Seasonal food consumption patterns and dietary diversity of rural preschool Ghanaian and Malawian children 2 3. *Ecology of food and nutrition* **29**:219-234.

Fuge R and Johnson CC (2015) Iodine and human health, the role of environmental geochemistry and diet, a review. *Applied Geochemistry* **63**:282-302.

Gayer J and Smith G (2015) Micronutrient fortification of food in southeast Asia: recommendations from an expert workshop. *Nutrients* **7**:646-658.

Gernand AD, Schulze KJ, Stewart CP, West K and Christian P (2016) Micronutrient deficiencies in pregnancy worldwide: health effects and prevention. *Nature reviews Endocrinology*.

GSS (2009) Ghana Demographic and Health Survey 2008., pp 1-483, Ghana Statistical Service - GSS, Ghana Health Service - GHS, and ICF Macro.

GSS (2011) Ghana Multiple Indicator Cluster Survey with an Enhanced Malaria Module and Biomarker Final Report: Monitoring the situation of children and women, pp 52-60, Ghana Statistical Service.
GSS (2014) Ghana living standards survey round 6 (GLSS 6) - main report, pp 16-20, Ghana Statistical Service.
GSS (2015) Ghana Demographic and Health Survey: Key Indicators 2014, pp 1-45, Ghana Statistical Service- GSS, GHS, and ICF Macro

Haider BA, Olofin I, Wang M, Spiegelman D, Ezzati M and Fawzi WW (2013) Anaemia, prenatal iron use, and risk of adverse pregnancy outcomes: systematic review and meta-analysis. *Bmj* **21**.
Haldimann M, Bochud M, Burnier M, Paccaud F and Dudler V (2015) Prevalence of iodine inadequacy in Switzerland assessed by the estimated average requirement cut-point method in relation to the impact of iodized salt. *Public health nutrition* **18**:1333-1342.
Hoddinott J, Yohannes, J., (2002) Dietary diversity as a food security indicator. (Discussion paper 136) pp 1-2, International Food Policy Research Institute (IFPRI). Washington D.C. .
Hoffmann K, Schulze MB, Schienkiewitz A, Nöthlings U and Boeing H (2004) Application of a new statistical method to derive dietary patterns in nutritional epidemiology. *American Journal of Epidemiology* **159**:935-944.
Hollowell JG, Staehling NW, Hannon WH, Flanders DW, Gunter EW, Maberly GF, Braverman LE, Pino S, Miller DT and Garbe PL (1998) Iodine nutrition in the United States. Trends and public health implications: iodine excretion data from National Health and Nutrition Examination Surveys I and III (1971–1974 and 1988–1994). *The Journal of Clinical Endocrinology & Metabolism* **83**:3401-3408.
Humphries D, Mosites E, Otchere J, Twum WA, Woo L, Jones-Sanpei H, Harrison LM, Bungiro RD, Benham-Pyle B and Bimi L (2011) Epidemiology of hookworm infection in Kintampo North Municipality, Ghana: patterns of malaria coinfection, anemia, and albendazole treatment failure. *The American journal of tropical medicine and hygiene* **84**:792-800.
IFPRI (2014) Global Nutriton Report: Ghana 2014 Nutrition Country Profile pp 1-2, International Food Policy Research Institute.
Jones AD, Shrinivas A and Bezner-Kerr R (2014) Farm production diversity is associated with greater household dietary diversity in Malawi: Findings from nationally representative data. *Food Policy* **46**:1-12.
Kennedy G, Nantel G and Shetty P (2003) The scourge of" hidden hunger": global dimensions of micronutrient deficiencies. *Food Nutrition and Agriculture*:8-16.
Kennedy GL, Pedro MR, Seghieri C, Nantel G and Brouwer I (2007) Dietary diversity score is a useful indicator of micronutrient intake in non-breast-feeding Filipino children. *The Journal of Nutrition* **137**:472-477.
Kontic-Vucinic O, Sulovic N and Radunovic N (2006) Micronutrients in women's reproductive health: II. Minerals and trace elements. *Int J Fertil Womens Med* **51**:116-124.
Korkalo L, Erkkola M, Heinonen AE, Freese R, Selvester K and Mutanen M (2016) Associations of dietary diversity scores and micronutrient status in adolescent Mozambican girls. *European journal of nutrition*:1-11.
Lorenzana PA and Mercado C (2002) Measuring household food security in poor Venezuelan households. *Public health nutrition* **5**:851-857.
Lorenzana PA and Sanjur D (1999) Abbreviated measures of food sufficiency validly estimate the food security level of poor households: measuring household food security. *J Nutr* **129**:687-692.
Lynch SR (2011) Why nutritional iron deficiency persists as a worldwide problem. *J Nutr* **141**:763S-768S.

Lynch SR and Cook JD (1980) Interaction of vitamin C and iron. *Ann N Y Acad Sci* **355**:32-44.

Malhotra N, Upadhyay RP, Bhilwar M, Choy N and Green T (2014) The role of maternal diet and iron-folic acid supplements in influencing birth weight: evidence from India's National Family Health Survey. *J Trop Pediatr* **60**:454-460.

Micke GC, Sullivan TM, Kennaway DJ, Hernandez-Medrano J and Perry VE (2014) Maternal endocrine adaptation throughout pregnancy to nutrient manipulation: Consequences for sexually dimorphic programming of thyroid hormones and development of their progeny. *Theriogenology* **4**:604-615.

Mirmiran P, Azadbakht L, Esmaillzadeh A and Azizi F (2004) Dietary diversity score in adolescents-a good indicator of the nutritional adequacy of diets: Tehran lipid and glucose study. *Asia Pacific journal of clinical nutrition* **13**:56-60.

Moursi MM, Arimond M, Dewey KG, Trèche S, Ruel MT and Delpeuch F (2008) Dietary diversity is a good predictor of the micronutrient density of the diet of 6-to 23-month-old children in Madagascar. *The Journal of Nutrition* **138**:2448-2453.

Nguyen PH, Avula R, Ruel MT, Saha KK, Ali D, Tran LM, Frongillo EA, Menon P and Rawat R (2013) Maternal and child dietary diversity are associated in Bangladesh, Vietnam, and Ethiopia. *The Journal of Nutrition* **143**:1176-1183.

Ogle BM, Hung PH and Tuyet HT (2001) Significance of wild vegetables in micronutrient intakes of women in Vietnam: an analysis of food variety. *Asia Pac J Clin Nutr* **10**:21-30.

Onyango A, Koski KG and Tucker KL (1998) Food diversity versus breastfeeding choice in determining anthropometric status in rural Kenyan toddlers. *Int J Epidemiol* **27**:484-489.

Palermo T, Rawat R, Weiser SD and Kadiyala S (2013) Food access and diet quality are associated with quality of life outcomes among HIV-infected individuals in Uganda. *PLoS ONE* **8**:e62353.

Parker ME, Mosites E, Reider K, Ndayishimiye N, Waring M, Nyandimbane G, Masumbuko D, Ndikuriyo L and Matthias D (2015) A Blinded, Cluster-Randomized, Placebo-Controlled School FeedingTrial in Burundi Using Rice Fortified With Iron, Zinc, Thiamine, and Folic Acid. *Food and nutrition bulletin*:0379572115615234.

Pearce EN, Andersson M and Zimmermann MB (2013) Global iodine nutrition: Where do we stand in 2013? *Thyroid : official journal of the American Thyroid Association* **23**:523-528.

Qureshi ME, Dixon J and Wood M (2015) Public policies for improving food and nutrition security at different scales. *Food Security* **7**:393-403.

Radlowski EC and Johnson RW (2013) Perinatal iron deficiency and neurocognitive development. *Front Hum Neurosci* **7**:00585.

Ross AC, Caballero B, Cousins RJ, Tucker KL and Ziegler TR (2014) *Modern Nutrition in Health and Disease*.

Ruel MT (2003a) Is dietary diversity an indicator of food security or dietary quality? A review of measurement issues and research needs. *Food Nutr Bull* **24**:231-232.

Ruel MT (2003b) Operationalizing Dietary Diversity: A Review of Measurement Issues and Research Priorities. *Journal of Nutrition* **133**:3911s - 3926s.

Ruel MT (2003c) Operationalizing dietary diversity: a review of measurement issues and research priorities. *The Journal of Nutrition* **133**:3911S-3926S.

Schmidt RJ, Tancredi DJ, Krakowiak P, Hansen RL and Ozonoff S (2014) Maternal intake of supplemental iron and risk of autism spectrum disorder. *Am J Epidemiol* **180**:890-900.

Stadlmayr B, Charrondiere UR, Addy P, Samb B, Enujiugha VN, Bayili RG, Fagbohoun EG, Smith IF, Thiam I and Burlingame B (2010) Composition of selected foods from West Africa. *Food and Agriculture Organization, Rome*:13-14.

Stewart CP, Iannotti L, Dewey KG, Michaelsen KF and Onyango AW (2013) Contextualising complementary feeding in a broader framework for stunting prevention. *Maternal and Child Nutrition* **9**:27-45.
Steyn N, Nel J, Nantel G, Kennedy G and Labadarios D (2006) Food variety and dietary diversity scores in children: are they good indicators of dietary adequacy? *Public health nutrition* **9**:644-650.
Strauss RS and Dietz WH (1997) Effects of intrauterine growth retardation in premature infants on early childhood growth. *The Journal of Pediatrics* **130**:95-102.
Tarini A, Bakari S and Delisle H (1999) [The overall nutritional quality of the diet is reflected in the growth of Nigerian children]. *Sante* **9**:23-31.
Tetens I, Bendtsen KM, Henriksen M, Ersbøll AK and Milman N (2007) The impact of a meat-versus a vegetable-based diet on iron status in women of childbearing age with small iron stores. *European journal of nutrition* **46**:439-445.
Torheim LE, Ouattara F, Diarra MM, Thiam FD, Barikmo I, Hatloy A and Oshaug A (2004) Nutrient adequacy and dietary diversity in rural Mali: association and determinants. *Eur J Clin Nutr* **58**:594-604.
UN UNS- (2008) Fact sheets on Nutrition and Food Security Indicators/Measures:Dietary Diversity (DD), pp 1-6, United Nations System.
Valera-Gran D, Garcia de la Hera M, Navarrete-Munoz EM, Fernandez-Somoano A, Tardon A, Julvez J, Forns J, Lertxundi N, Ibarluzea JM, Murcia M, Rebagliato M and Vioque J (2014) Folic acid supplements during pregnancy and child psychomotor development after the first year of life. *JAMA Pediatr* **168**:e142611.
Wang X, Lan X, Radunz AE and Khatib H (2015) Maternal nutrition during pregnancy is associated with differential expression of imprinted genes and DNA methyltranfereases in muscle of beef cattle offspring. *J Anim Sci* **93**:35-40.
Weiser SD, Hatcher A, Frongillo EA, Guzman D, Riley ED, Bangsberg DR and Kushel MB (2013) Food insecurity is associated with greater acute care utilization among HIV-infected homeless and marginally housed individuals in San Francisco. *Journal of general internal medicine* **28**:91-98.
WFP (2012) Comprehensive Food Security & Vulnerability Analysis - Focus on Northern Ghana, pp 1-144, World Food Programme.
WHO (2001) Iron deficiency anaemia: assessment, prevention and control: a guide for programme managers, pp 15-18, World Health Organization, Geneva.
World Health Organization (2011) Serum ferritin concentrations for the assessment of iron status and iron deficiency in populations.
Zhao J, Xu F, Zhang Q, Shang L, Xu A, Gao Y, Chen Z, Sullivan KM and Maberly GF (1999) Randomized clinical trial comparing different iodine interventions in school children. *Public health nutrition* **2**:173-178.

APPENDICES

Appendix A: Nutrient composition of staple foods in the Upper East Region of Ghana

Usual household foods consumed in the Upper East Region of Northern Ghana

Food group/item	*Frequency/Weight (Kubuga, 2015, unpublished)	**Micronutrients (Stadlmayr et al., 2010)				
**Cereals**	**Consumption**	**Energy (kcal)**	**Fe (mg/100g)**	**Vit C (mg/100g)**	**Zn (mg/100g)**	**Se & I (mg/100g)**
Maize	Main	136-356	1.1 - 3.0	0	0.58-0.63	Unknown
millet	Main	145-396	3.9 - 9.5	0	1.47-2.98	Unknown
Rice	Sparingly/depends on availability	142-353	0.7-1	0	1.0-2.02	Unknown
**Legumes and pulses**						
Boil pigeon beans	Main	109 -111	1.5	Tr	1 - 1.26	Unknown
Boil cow pea	Main	111-118	2.7	0.2 - 0.8	1.1-1.54	Unknown
Ground nut	Main	586	2.3 - 4	0	2.6	Unknown
Dawadawa (fermented African Locust Bean)	Main	445	33.2	6	Unknown	Unknown
Nieri / Agushie/mellon seeds	Sparingly/ availability	593	6.1	Tr	7.12	Unknown
Sesame seeds	Sparingly/ availability	577	11	Tr	7.75	Unknown
Raw bambara beans	Sparingly/Mainly for funerals	141-369	3-6 (raw)		0.8-3.36	Unknown
**Root and tubers**						
Sweet potatoe	Seasonal	80-115	0.8-1.1	15-22.9	0.31-0.35	Unknown
African potato	Seasonal					Unknown
**Vegetables (predominant in rainy season)**						
Hibiscus cannabinus	Main (scarce in dry season)	42	3.2 - 12	14 - 20	0.71	Unknown
Hibiscus sabdariffa	Main (scarce in dry season)	42	3.2 - 12	14 - 20	0.71	Unknown
Amaranth leaves	Sparingly (scarce in dry season)	39	4.9	19	0.2-0.9	Unknown
Beans leaves	Seasonal					Unknown
Okra fruit	Main	33	0.8 -1	19	0.6	
Okra leaves	Seasonal	43-62	0.5 -3.5	15.2-34	0.35-0.7	Unknown
Boabab leaves	main (wild)	74	3.1	20	0.7	Unknown
Wild leaves	Coping with shortages					Unknown
**Meat and animal products**						
Poultry/birds/beef/mouton/etc	Occasional	134-209	1 - 3.5	0	1.36-6	Unknown
Game	Occasional	330	9.9	0	6.06	Unknown
Amani (fish)	Occasional	29.1				

NB: Foods and ingredients of usual and habitual meals of households in the Upper East Region of Northern Ghana

Appendix B. Dyad Consent Form-Intervention

(Mother & Child) - Intervention

**Mother's code**			
**Child's code**			

Community interventions to improve iron and iodine status in mother and child dyads in northern Ghana

Introduction/purpose

You and your child are invited to participate in this research work which aims at contributing to reducing malnutrition in Ghana by developing noninvasive and dietary approaches for trace minerals (iron iodine and zinc) status screening at population levels. These nutrients are of public health concern because of their biological roles in healthy body functions and development. Deficiencies lead to devastating and irreparable damages such as mental and growth impairments. At the moment, measurement of these minerals are done biochemically which are not easy to carry out in developing countries like Ghana. Nutritional assessment in these countries is challenging much due to absence of food composition datasets of native regional foods and nutritional status impacted by various non-dietary and environmental factors such as infectious diseases. It is therefore very vital to investigate the association between trace minerals biomarkers status and dietary and household characteristics to contribute to an easier, quicker and noninvasive approaches to predicting population based nutrients deficiencies for appropriate interventions.

Study procedure, confidentiality and voluntariness

If you agree to participate, you would be engaged in 1) an interview on your household characteristics, 2) a recall of foods eaten the previous day by both you and your child, 3) giving 5ml of blood samples from both you and your child's subcutaneous veins, 4) 5mls of urine samples, and 5) 5g of feces. We will re-schedule time period convenient for you and your child for feces samples when you are not able to provide samples at the time of interview. No names will be attached to samples as such no one else will be able to identify your samples after they leave your house. Given respondent's code would only be used for data analysis and will not be known to a third party except the research team. The results will be kept strictly confidential and will not be shared with anyone other than members of our research team. No additional analysis would be done on your samples or left over samples other than the stated purpose. Samples will be taken four times: at week 0 (baseline) and 12 (followup1) in phase 1 (this dry season) and in phase 2 (next dry season), we will collect the same samples at week 0 and 12 again. Each interview would take 40 - 60 minutes of your time. You and your child would be given a token of 10 GHS each for your participation at each interview. You and your child would be provided with 2-3 meals/week of hibiscus sabdariffa in phase 1. In addition, you would be provided with containers and supported to raise vegetables during each phase. Your research records would be kept by the department of community nutrition, University for Development Studies and unused samples stored by MSU for three years and discarded appropriately thereafter following the terms for Material Transfer Agreement (MTA) of MSU Technologies. NHRCIRB will not have access to your research records.

Benefits/risks

- Drawing of blood from the subcutaneous vain of both you and your child is not expected to cause any health complications. The needles and syringes are sterile and not re-usable, disease transfer from one person to another is thus avoided. However, the risks associated with blood drawing include very minor pain, at the point of needle insertion; bleeding, skin discoloration at point of needle insertion, and local infection. To control these, health professionals will be use in drawing blood. When you or your child is found to have parasites in the stool, have malaria, or is iron or iodine deficient, the health care worker on our research team will coordinate the required health care for you using your national health insurance package. You and your child's participation is completely voluntary. Refusal to participate will involve no penalty. You and or your child will be allowed to discontinue participation at any time without penalty. In addition to the remuneration for participation and the container gardening, your participation will help us to increase knowledge base on improving trace mineral status in mother-child (6-23 mo) dyads, additionally your participation will help us to develop dietary screening tools that will enable the provision of timely and good health services by health providers to community members. This would also help stake holders in the health sector to better address challenges of trace minerals through policy making and national nutrition programs.

If you have any questions or concerns about your role and rights as a participants, or would like to make a complaint about this educational project please contact the student researcher, Advisor or NHRCIRB chairman. Additionally, by being a part of this study, if at any point you feel you are or have been hurt, please contact the student researcher (Clement Kubuga) or the chairman of the NHRCIRB through the contact information provided below:

1. **Student Researcher:** Clement Kubuga, FSHN, Michigan State University. E-mail: kubugacl@msu.edu Phone: 0203820163
2. **Student's Advisors:** Won O. Song, PhD, MPH, RD, FSHN, Michigan State University E-mail: song@msu.edu , Phone: +1 517-353-3332
3. **Chairman,** NHRCIRB through the IRB Administrator on telephone (+233) 020-166-0158 or irb@navrongo-hrc.org

To agree to be a part of this study, sign and write your initials in the corresponding line (s) below or thump print in the corresponding line:

I have read and understood what the study entails and agree that my child and I to participate in this study by responding to a dietary and household survey and providing urine and blood samples as stipulated for this study.

______________________	______________________	__________
Participant Initials (please print)	Participant Signature	Date

*The purpose and approaches of the study have been explained to me in languages

that I understand. I and my child agree to participate in this study and to provide our urine and blood samples and give my (our) consent to have our samples used as stipulated for this study.

______________________________ ______________________
Thumbprint (Participant) Date
*for persons who cannot read and write

Thank you for your participation!

Appendix C: Dyad Consent Form-Control

(Mother & child) - Control

Mother's code			
Child's code			

Community interventions to improve iron and iodine status in mother and child dyads in northern Ghana

Introduction/purpose

You and your child are invited to participate in this research work which aims at contributing to reducing malnutrition in Ghana by developing noninvasive and dietary approaches for trace minerals (iron iodine and zinc) status screening at population levels. These nutrients are of public health concern because of their biological roles in healthy body functions and development. Deficiencies lead to devastating and irreparable damages such as mental and growth impairments. At the moment, measurement of these minerals are done biochemically which are not easy to carry out in developing countries like Ghana. Nutritional assessment in these countries is challenging much due to absence of food composition datasets of native regional foods and nutritional status impacted by various non-dietary and environmental factors such as infectious diseases. It is therefore very vital to investigate the association between trace minerals biomarkers status and dietary and household characteristics to contribute to an easier, quicker and noninvasive approaches to predicting population based nutrients deficiencies for appropriate interventions.

Study procedure, confidentiality and voluntariness

If you agree to participate, you would be engaged in 1) an interview on your household characteristics, 2) a recall of foods eaten the previous day by both you and your child, 3) giving 5ml of blood samples from both you and your child's subcutaneous veins, 4) 5mls of urine samples, and 5) 5g of feces. We will re-schedule time period convenient for you and your child for feces samples when you are not able to provide samples at the time of interview. No names will be attached to samples as such no one else will be able to identify your samples after they leave your house. Given respondent's code would only be used for data analysis and will not be known to a third party except the research team. The results will be kept strictly confidential and will not be shared with anyone other than members of our research team. No additional analysis would be done on your samples or left over samples other than the stated purpose. Samples will be taken two times: at week 0 (baseline) and 12 (followup1). Each interview would take 40 - 60 minutes of your time. Each interview would take 40 - 60 minutes of your time. You and your child would be given a token of 10 GHS each for your participation at each interview. Your research records would be kept by the department of community nutrition, University for Development Studies and unused samples stored by MSU for three years and discarded appropriately thereafter following the terms for Material Transfer Agreement (MTA) of MSU Technologies. NHRCIRB will not have access to your research records.

Benefits/risks

- Drawing of blood from the subcutaneous vain of both you and your child is not expected to cause any health complications. The needles and syringes are sterile and not re-usable, disease transfer from one person to another is thus avoided. However, the risks associated with blood drawing include very minor pain, at the point of needle insertion; bleeding, skin discoloration at point of needle insertion, and local infection. To control these, health professionals will be use in drawing blood. When you or your child is found to have parasites in the stool, have malaria, or is iron or iodine deficient, the health care worker on our research team will coordinate the required health care for you using your national health insurance package. You and your child's participation is completely voluntary. Refusal to participate will involve no penalty. You and or your child will be allowed to discontinue participation at any time without penalty. In addition to the remuneration, your participation will help us to increase knowledge base on improving trace mineral status in mother-child (6-23 mo) dyads. Additionally your participation will help us to develop dietary screening tools that will enable the provision of timely and good health services by health providers to community members. This would also help stake holders in the health sector to better address challenges of trace minerals through policy making and national nutrition programs.

If you have any questions or concerns about your role and rights as a participants, or would like to make a complaint about this educational project please contact the student researcher, Advisor or NHRCIRB chairman. If at any point you feel you are or have been hurt please contact the student researcher (Clement Kubuga) or the chairman of the NHRCIRB through the contact information provided below:

4. **Student Researcher:** Clement Kubuga, FSHN, Michigan State University. E-mail: kubugacl@msu.edu Phone: 0203820163
5. **Student's Advisors:** Won O. Song, PhD, MPH, RD, FSHN, Michigan State University E-mail: song@msu.edu , Phone: +1 517-353-3332
6. **Chairman,** NHRCIRB through the IRB Administrator on telephone (+233) 020-166-0158 or irb@navrongo-hrc.org

To agree to be a part of this study, sign and write your initials in the corresponding line (s) below or thump print in the corresponding line:

I have read and understood what the study entails and agree that my child and I to participate in this study by responding to a dietary and household survey and providing urine and blood samples as stipulated for this study.

Participant Initials (please print)	Participant Signature	Date

*The purpose and approaches of the study have been explained to me in languages that I understand. I and my child agree to participate in this study and to provide our urine and blood samples and give my (our) consent to have our samples used as stipulated for this study.

Thumbprint (Participant)	Date

*for persons who cannot read and write

Thank you for your participation!

Appendix D: Parental Consent Form-Intervention

(Parental consent for minor mother) - Intervention

Respondent's code			
Minor mother's code			
Child's code			

Community interventions to improve iron and iodine status in mother and child dyads in northern Ghana

Introduction/purpose

Your child (minor mother) and grandchild are invited to participate in this research work which aims at contributing to reducing malnutrition in Ghana by developing noninvasive and dietary approaches for trace minerals (iron iodine and zinc) status screening at population levels. These nutrients are of public health concern because of their biological roles in healthy body functions and development. Deficiencies lead to devastating and irreparable damages such as mental and growth impairments. At the moment, measurement of these minerals are done biochemically which are not easy to carry out in developing countries like Ghana. Nutritional assessment in these countries is challenging much due to absence of food composition datasets of native regional foods and nutritional status impacted by various non-dietary and environmental factors such as infectious diseases. It is therefore very vital to investigate the association between trace minerals biomarkers status and dietary and household characteristics to contribute to an easier, quicker and noninvasive approaches to predicting population based nutrients deficiencies for appropriate interventions.

Study procedure, confidentiality and voluntariness

If you agree for your daughter and grandchild to participate, your daughter would be engaged in 1) an interview on her household characteristics, 2) a recall of foods eaten previous day both her and her child 3) giving 5ml of blood samples from the subcutaneous veins of both her and her child, 4) 5mls of urine samples and 5) 5g of feces from her and her child. We will re-schedule time period convenient for feces samples when they are not able to provide samples at the time of interview. No names will be attached to samples as such no one else will be able to identify their samples after they leave your house. Given respondent's code would only be used for data analysis and will not be known to a third party except the research team. The results will be kept strictly confidential and will not be shared with anyone other than members of our research team. No additional analysis would be done on their samples or left over samples other than the stated purpose. Samples will be taken four times: at week 0 (baseline) and 12 (followup1) in phase 1 (this dry season) and in phase 2 (next dry season), we will collect the same samples at week 0 and 12 again. Each interview would take 40 - 60 minutes of their time. Your children would be given a token of 10 GHS each for their participation at each interview. They would be provided with 2-3 meals/week of hibiscus sabdariffa in phase 1. In addition, your daughter would be

provided with containers and supported to raise vegetables during each phase. Their research records would be kept by the department of community nutrition, university for development studies and unused samples store by MSU for three years and discarded appropriately thereafter following the terms for Material Transfer Agreement (MTA) of MSU Technologies. NHRCIRB will not have access to their research records.

Benefits/risks

- Drawing of blood from the subcutaneous vain of both your daughter and grandchild is not expected to cause any health complications. The needles and syringes are sterile and not re-usable, disease transfer from one person to another is thus avoided. However, the risks associated with blood drawing include very minor pain, at the point of needle insertion; bleeding, skin discoloration at point of needle insertion, and local infection. To avoid these health professionals will be use in drawing blood. When either your daughter or grandchild is found to have parasites in their stool, have malaria, or are iron or iodine deficient, the health care worker on our research team will coordinate the required health care for them using their national health insurance package. Your daughter and grandchild's participation are completely voluntary. Refusal to participate will involve no penalty. They will be allowed to discontinue participation at any time without penalty. In addition to the remuneration for participation and the container gardening, their participation will help us to increase knowledge base on improving trace mineral status in mother-child (6-23 mo) dyads, additionally your participation will help us to develop dietary screening tools that will enable the provision of timely and good health services by health providers to community members. This would also help stake holders in the health sector to better address challenges of trace minerals through policy making and national nutrition programs.

If you have any questions or concerns about your children's roles and rights as a participants, or would like to make a complaint about this educational project please contact the student researcher, Advisor or NHRCIRB chairman. Additionally, by being a part of this study, if at any point you feel your children are or have been hurt, please contact the student researcher (Clement Kubuga) or the chairman of the NHRCIRB through the contact information provided below:

1. **Student Researcher:** Clement Kubuga, FSHN, Michigan State University. E-mail: kubugacl@msu.edu Phone: 0203820163
2. **Student's Advisors:** Won O. Song, PhD, MPH, RD, FSHN, Michigan State University E-mail: song@msu.edu , Phone: +1 517-353-3332
3. **Chairman,** NHRCIRB through the IRB Administrator on telephone (+233) 020-166-0158 or irb@navrongo-hrc.org

To agree for *your child* to be a part of this study, sign and write your initials in the corresponding line (s) below or thumb print in the corresponding line:

I have read and understood what the study entails and agree for my daughter and grandchild to participate in this study by responding to a dietary and household survey and providing their urine, feces and blood samples as stipulated for this study.

____________________ ____________________ _________

Participant Initials (please print) Participant Signature Date

*The purpose and approaches of the study have been explained to me in languages that I understand. I agree for *my daughter and grandchild* to participate in this study and to provide their urine, feces and blood samples and give my consent to have their samples used as stipulated for this study.

Thumbprint (Participant) Date

*for persons who cannot read and write

Thank you for your participation!

Appendix E: Parental Consent Form-Control

(Parental consent for minor mother) - Control

Respondent's code			
Minor mother's code			
Child's code			

Community interventions to improve iron and iodine status in mother and child dyads in northern Ghana

Introduction/purpose

Your child (minor mother) and grandchild are invited to participate in this research work which aims at contributing to reducing malnutrition in Ghana by developing noninvasive and dietary approaches for trace minerals (iron iodine and zinc) status screening at population levels. These nutrients are of public health concern because of their biological roles in healthy body functions and development. Deficiencies lead to devastating and irreparable damages such as mental and growth impairments. At the moment, measurement of these minerals are done biochemically which are not easy to carry out in developing countries like Ghana. Nutritional assessment in these countries is challenging much due to absence of food composition datasets of native regional foods and nutritional status impacted by various non-dietary and environmental factors such as infectious diseases. It is therefore very vital to investigate the association between trace minerals biomarkers status and dietary and household characteristics to contribute to an easier, quicker and noninvasive approaches to predicting population based nutrients deficiencies for appropriate interventions.

Study procedure, confidentiality and voluntariness

If you agree for your daughter and grandchild to participate, your daughter would be engaged in 1) an interview on her household characteristics, 2) a recall of foods eaten previous day both her and her child 3) giving 5ml of blood samples from the subcutaneous veins of both her and her child, 4) 5mls of urine samples and 5) 5g of feces from her and her child. We will re-schedule time period convenient for feces samples when they are not able to provide samples at the time of interview. No names will be attached to samples as such no one else will be able to identify their samples after they leave your house. Given respondent's code would only be used for data analysis and will not be known to a third party except the research team. The results will be kept strictly confidential and will not be shared with anyone other than members of our research team. No additional analysis would be done on their samples or left over samples other than the stated purpose. Samples will be taken two times: at week 0 (baseline) and 12 (followup1). Each interview would take 40 - 60 minutes of your time. Each interview would take 40 - 60 minutes of their time. Your children would be given a token of 10 GHS each for their participation at each interview. Their research records would be kept by the department of community nutrition, University for Development Studies and unused samples stored by MSU for three years and discarded

appropriately thereafter following the terms for Material Transfer Agreement (MTA) of MSU Technologies. NHRCIRB will not have access to your research records.

Benefits/risks

- Drawing of blood from the subcutaneous vain of both your daughter and grandchild is not expected to cause any health complications. The needles and syringes are sterile and not re-usable, disease transfer from one person to another is thus avoided. However, the risks associated with blood drawing include very minor pain, at the point of needle insertion; bleeding, skin discoloration at point of needle insertion, and local infection. To avoid these health professionals will be use in drawing blood. When either your daughter or grandchild is found to have parasites in their stool, have malaria, or are iron or iodine deficient, the health care worker on our research team will coordinate the required health care for them using their national health insurance package. Your daughter and grandchild's participation are completely voluntary. Refusal to participate will involve no penalty. They will be allowed to discontinue participation at any time without penalty. In addition to the remuneration, your participation will help us to increase knowledge base on improving trace mineral status in mother-child (6-23 mo) dyads. Additionally your children's participation will help us to develop dietary screening tools that will enable the provision of timely and good health services by health providers to community members. This would also help stake holders in the health sector to better address challenges of trace minerals through policy making and national nutrition programs.

If you have any questions or concerns about your children's role and rights as a participants, or would like to make a complaint about this educational project please contact the student researcher, Advisor or NHRCIRB chairman. If at any point you feel your children are or have been hurt please contact the student researcher (Clement Kubuga) or the chairman of the NHRCIRB through the contact information provided below:

- **Student Researcher:** Clement Kubuga, FSHN, Michigan State University. E-mail: kubugacl@msu.edu Phone: 0203820163
- **Student's Advisors:** Won O. Song, PhD, MPH, RD, FSHN, Michigan State University E-mail: song@msu.edu , Phone: +1 517-353-3332
- **Chairman,** NHRCIRB through the IRB Administrator on telephone (+233) 020-166-0158 or irb@navrongo-hrc.org

To agree for *<u>your child</u>* to be a part of this study, sign and write your initials in the corresponding line (s) below or thumb print in the corresponding line:

I have read and understood what the study entails and agree for my daughter and grandchild to participate in this study by responding to a dietary and household survey and providing their urine, feces and blood samples as stipulated for this study.

______________________________	_________________________	_____________
Participant Initials (please print)	Participant Signature	Date

*The purpose and approaches of the study have been explained to me in languages

that I understand. I agree for *my daughter and grandchild* to participate in this study and to provide their urine, feces and blood samples and give my consent to have their samples used as stipulated for this study.

______________________________ ________________________

Thumbprint (Participant) Date

*for persons who cannot read and write

Thank you for your participation!

Appendix F: Questionnaire

Michigan State University

Department of Food Science and Human Nutrition

Community interventions to improve iron and iodine status in mother and child dyads in northern Ghana

Questionnaire ID………………

Background information

1. Name of interviewer……………… 2. Date …………… 3. Community ………… 4. Mother's ID………………

5. Age of mother (yrs) ……………. 6. Weight of Mother (kg)………….. 7. Height of mother (m) …………

8. Mother Hb (g/l) ……… sFe (ug/l) ……….sTfR (nmol/l) ..…… 9. Religion…………. 10. Ethnicity……………….

9. Education: a. none ☐ b. Primary ☐ c. Secondary ☐ d. college/tertiary ☐

10. Mother's occupation……………………………. *Mother's malaria status……………………………..

Marital status: a. married ☐ b. Single ☐ c. divorced/separated ☐

11. Child's age (months) ………… 12. Child's height (m) ………… 13. Child's weight (kg)…………

14. Child's Hb (g/l) ……………… sFe (ug/l)……… sTfR (nmol/l) ……

Number of siblings…………….. *Child's malaria status…………….

Household characteristics

15. Sex of household head ………… 16. Age of Household head ………… 17. No of adults in household ……

18. Number of Children <5yrs ………………… 19. Building type ………………… 20. Source of water ………………

21. Type of toilet……………………… 22 Lives with animals in same yard ……..………………..

23. Does your household has any of the under listed items? (Please tick in check box when present)

a. Bicycle ☐ b. Motorbike ☐ c. Clock ☐ d. Radio ☐ e. Sewing Machine ☐ f. Bed ☐ g. Table ☐
h. Cabinet/Cupboard ☐ i. Mobile Phone ☐ j. Refrigerator ☐ k. Generator/Invertor ☐ l. Television ☐
m. Video Deck ☐ n. Dvd/Vcd ☐ o. Electricity ☐ p. Washing Machine ☐ q. Computer ☐
r. Digital Camera ☐ s. Non-Digital Camera ☐ t. car ☐ u. tractor ☐

18. Does this household own the under listed (If yes to any, input the corresponding numbers in the 1st column – N*)	N*	1	2	3
a. Cattle				
b. Goat				
a. Sheep				
b. Pigs				
c. Rabits				
d. Grasscutter				
e. Chicken				
f. Guinea fowls				
g. other poultry				
N* = input number of animals, 1 = none, 2 = more than 95, 3 = don't know				

Women empowerment

19. Who usually	1	2	3
a. decides how the money you earn will be used?			
b. decides how your husband's/partner's earnings will be used?			
c. makes decisions about health care for yourself			
d. makes decisions about making major household purchases?			
e. makes decisions about making purchases for daily household needs?			
f. makes decisions about visits to your family or relatives?			
g. makes decisions about how many children to have?			
Key: 1 = you, 2 = partner, 3 = both			

20. Who earns more money in your household? a. you ☐ b. partner ☐ c. about same ☐

Appendix E:

Average composition of *hibiscus S* soup

Item	Item Qty (g)	Fe (mg) qty/serving	Kcal	Protein Qty/serving	Item Qty (g)	Fe (mg) qty/serving	kcal	Protein Qty/serving	Item Qty (g)	Fe (mg) qty/serving	kcal	Protein Qty/serving
Veo	500	17.3			200	12.1			516.4	8.1		
Amani	100	8.3			50	7.3			45.4	1.7		
dawadawa	50	4.7			40	6.6			0			

G.Nut	150	0.6			120	2.4			227	1.2			
Total	7 L	**30.9***			4 L	**28.4***			15.5 L	**10.9***			

Usual servings is two ladles (2L) in each case. *Serving size is two ladles
Veo (Hibiscus Saddarifa), Amani (dried fish, herrings), dawadawa (African Locust bean), G.nut (groundnut).
Rural consumption: Women (3L – 5L), children (0.25L – 2L)

24 HR Recall Questionnaire
Option A

I would like to ask you about liquids or foods that (NAME……...)/you may have had yesterday during the day or at night. I am interested in whether your child/you had the item even if it was combined with other foods.										
Did (NAME ………...)/you drink (eat)	Child					Mother				
	Yes	No	DK	Freq	Qty (g)	Yes	No	DK	Freq	Qty (g)
1. Milk such as tinned, powdered, or fresh animal milk?										
2. Tea or coffee?										
3. Any other liquids (juice, cocoa)?										
4. Bread, rice, noodles, or other foods made from grains (kenkey, banku, koko,tuo zaafi, akple, weanimix)?										
5. Pumpkin, red or yellow yams, carrots, sweet potatoes that are yellow or orange inside?										
6. White potatoes, white yams, manioc, cassava, cocoyam, fufu or any other foods made from roots, tubers or plantain?										
7. Any dark green, leafy vegetables (kontomire, aleefu, ayoyo, kale, cassava leaves)?										
8. Ripe mangoes, pawpaw?										
9. Any other fruits or vegetables (e.g. bananas, avocados, tomatoes, oranges, apples)?										
10. Liver, kidney, heart or other organ meats?										
11. Any meat, such as beef, pork, lamb, goat, chicken, or duck?										
12. Eggs?										
13. Fresh or dried fish or shellfish (e.g. prawn, lobster)?										
14. Any foods made from beans, peas, lentils, or nuts?										
15. Cheese, yogurt or other milk products?										
16. Any oil, fats, or butter, or foods made with any of these?										
17. Any sugary foods such as chocolates, sweets, candies, pastries, cakes, or biscuits?										

***Option A* procedure:** Ask for what has been consumed the previous day without mentioning food items to avoid prejudice. Tick as foods are mentioned, probe for frequency and usual quantities in the past 7 days. Probe for other foods consumed within the week, their frequencies and usual quantities consumed. Probe for alcohol consumption, smoking and substance abuse

Option B

**24 hour recall ……………………………………………….**		**Day of the week:**	
**Time / period of day**	**quantity of food eaten**	**Detailed food description**	**Source of food**

Probe for alcohol consumption, smoking and substance abuse

Appendix G: MSU IRB Form

MICHIGAN STATE UNIVERSITY

Initial IRB Application Approval

May 3, 2016

To: Won Song
139 GM Trout Building

Re: **IRB**# 16-448 Category: EXPEDITED 2(a), 2(b), 3, 7
Approval Date: May 2, 2016
Expiration Date: May 1, 2017

Title: Community based agriculture project to increase trace minerals status in mother and child dyads in northern Ghana (CGA#125396)

The Institutional Review Board has completed their review of your project. I am pleased to advise you that **your project has been approved**.

The committee has found that your research project is appropriate in design, protects the rights and welfare of human subjects, and meets the requirements of MSU's Federal Wide Assurance and the Federal Guidelines (45 CFR 46 and 21 CFR Part 50). The protection of human subjects in research is a partnership between the IRB and the investigators. We look forward to working with you as we both fulfill our responsibilities.

Renewals: IRB approval is valid until the expiration date listed above. If you are continuing your project, you must submit an ***Application for Renewal*** application at least one month before expiration. If the project is completed, please submit an ***Application for Permanent Closure***.

Revisions: The IRB must review any changes in the project, prior to initiation of the change. Please submit an ***Application for Revision*** to have your changes reviewed. If changes are made at the time of renewal, please include an ***Application for Revision*** with the renewal application.

Problems: If issues should arise during the conduct of the research, such as unanticipated problems, adverse events, or any problem that may increase the risk to the human subjects, notify the IRB office promptly. Forms are available to report these issues.

Please use the IRB number listed above on any forms submitted which relate to this project, or on any correspondence with the IRB office.

Good luck in your research. If we can be of further assistance, please contact us at 517-355-2180 or via email at IRB@msu.edu. Thank you for your cooperation.

Sincerely,

Ashir Kumar, M.D.
BIRB Chair

c: Clement Kubuga

Office of Regulatory Affairs
Human Research Protection Programs

Biomedical & Health Institutional Review Board (BIRB)

Community Research Institutional Review Board (CRIRB)

Social Science Behavioral/Education Institutional Review Board (SIRB)

Olds Hall
408 West Circle Drive, #207
East Lansing, MI 48824
(517) 355-2180
Fax: (517) 432-4503
Email: irb@msu.edu
www.hrpp.msu.edu

MSU is an affirmative-action, equal-opportunity employer.

Appendix H: Ghana nutrition health and population statistics

2008 Ghana Demographic and Health Survey

The 2008 Ghana Demographic and Health Survey provides up-to-date information on the population and health situation in Ghana. The 2008 GDHS is the fifth national demographic and health survey conducted here. The survey contains information on fertility, family planning, maternal and child health and nutrition, childhood mortality, HIV/AIDS-related knowledge and behaviour, and domestic violence.

The survey is based on a nationally representative sample. It provides estimates for rural and urban areas and for the 10 regions.

**Coverage**	
Households interviewed	11,778
Women (15–49) interviewed	4,916
Men (15–59) interviewed	4,568
**Response rates**	
Household interview	99%
Women's interview	97%
Men's interview	96%

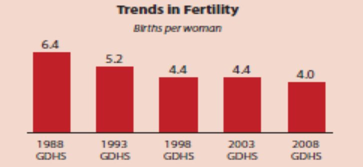

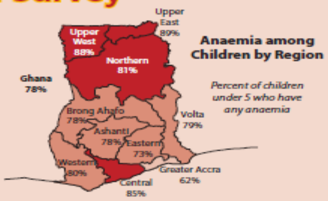

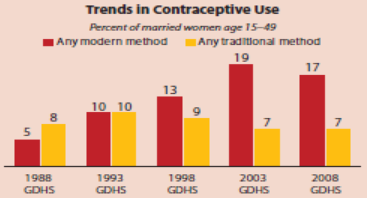

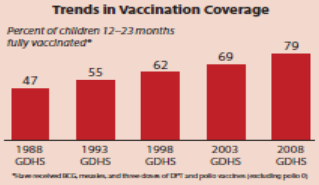

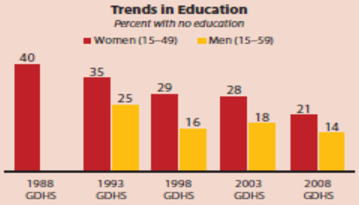

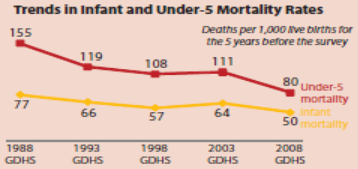

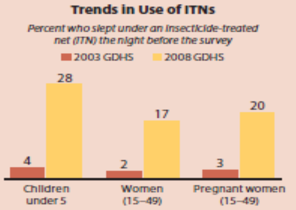

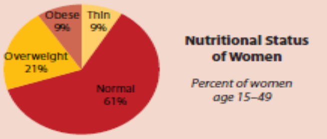

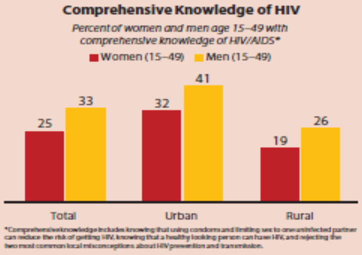

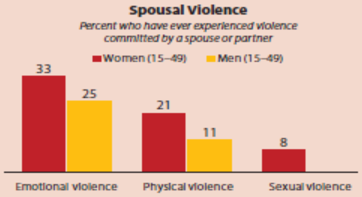

For more information about the 2008 GDHS, please contact:

In Ghana:
Ghana Statistical Service (GSS)
P.O. Box 1098
Accra, Ghana
Telephone: 233-21-671-732
Fax: 233-21-671-731

In USA:
MEASURE DHS
Macro International Inc.
11785 Beltsville Drive
Calverton, MD 20705 USA
Telephone: 301-572-0200; Fax: 301-572-0999
www.measuredhs.com
Email: reports@measuredhs.com

The 2008 Ghana Demographic and Health Survey (GDHS) was carried out by the Ghana Statistical Service and the Ghana Health Service. ICF Macro, an ICF International company, provided financial and technical assistance for the survey through the USAID-funded MEASURE DHS programme. Local costs for the survey were partially funded by: the Ministry of Health (MOH), the Ghana Statistical Service (GSS), the Ghana AIDS Commission (GAC), UNICEF, UNFPA, and Danida.

USAID Ghana Statistical Service unicef UNFPA Danida

2008 Ghana Demographic and Health Survey

Fact Sheet

Ghana

2008 GDHS

				Region									
**Fertility**	Total	Urban	Rural	Western	Central	Greater Accra	Volta	Eastern	Ashanti	Brong Ahafo	Northern	Upper East	Upper West
Total fertility rate (number of children per woman)	4.0	3.1	4.9	4.2	5.4	2.5	3.8	3.6	3.6	4.1	6.8	4.1	5.0
Women age 15–19 who are mothers or currently pregnant (%)	13	11	16	7	23	7	16	8	11	22	23	11	13
Median age at first marriage for women age 25–49 (years)	19.8	21.3	18.7	20.3	19.7	22.9	19.2	19.9	19.5	18.4	19.3	17.8	18.1
Median age at first sexual intercourse for women age 25–49 (years)	18.4	18.8	17.9	18.6	17.8	19.0	18.0	17.9	18.2	18.4	19.4	17.9	17.2
Median age at first birth for women age 25–49 (years)	20.7	22.1	20.0	21.0	20.2	23.2	20.4	20.2	20.8	20.2	20.7	19.5	19.5
Married women (age 15–49) wanting no more children (%)	37	37	36	45	41	46	39	47	35	26	20	34	24
**Family Planning (currently married women, age 15–49)**													
Women currently using any family planning method (%)	24	27	21	19	23	33	29	24	27	29	6	15	22
Women currently using any modern family planning method (%)	17	19	15	13	17	22	21	17	16	22	6	14	21
Women with an unmet need for family planning[1] (%)	35	32	38	39	50	27	34	40	37	35	32	32	28
**Maternal and Child Health**													
**Maternity care**													
Pregnant women who received antenatal care from a skilled provider[2] (%)	95	98	94	96	92	96	91	96	97	96	96	96	98
Births assisted by a skilled provider[2] (%)	59	84	43	62	54	84	54	61	73	66	27	47	46
Births delivered in a health facility (%)	57	82	42	58	52	84	54	59	70	65	26	46	45
Mothers who received first postnatal checkup within 2 days after delivery (%)	68	82	59	62	67	89	49	76	80	72	45	65	75
**Child immunization** Children 12–23 months fully vaccinated[3] (%)	79	78	79	82	(73)	(80)	(73)	76	85	(94)	59	(88)	89
**Nutrition**													
Children under 5 who are stunted (%)	28	21	32	27	34	14	27	38	27	25	32	36	25
Children under 5 who are wasted (%)	9	8	9	6	12	6	5	6	9	5	13	11	14
Children under 5 who are underweight (%)	14	11	16	10	17	7	14	9	12	14	22	27	13
Children under 5 who are overweight (%)	5	7	4	6	10	5	8	12	4	3	2	1	3
Children under 5 with any anaemia[4] (%)	78	68	84	80	85	62	79	73	78	78	81	89	88
Women age 15–49 with any anaemia[4] (%)	59	55	62	71	64	51	58	58	60	58	59	48	67
**Malaria**													
Households with at least 1 insecticide-treated net (ITN) (%)	33	27	38	38	30	20	40	33	29	46	27	47	46
Children under 5 who slept under an ITN the night before the survey (%)	28	24	31	36	19	17	40	36	25	50	11	37	34
Pregnant women who slept under an ITN the night before the survey (%)	20	13	25	(25)	(7)	(13)	(34)	*	17	*	7	(43)	(25)
**Childhood Mortality**													
Infant mortality (between birth and first birthday)[5]	50	49	56	51	(73)	(39)	(37)	(53)	58	(37)	70	(46)	97
Under-five mortality (between birth and fifth birthday)[5]	80	75	90	(65)	(108)	(50)	(50)	(81)	80	(76)	137	(78)	(142)
**HIV/AIDS-related Knowledge**													
Knows ways to prevent HIV and AIDS:													
Having one sexual partner (women age 15–49/men age 15–49) (%)	85/88	88/90	82/87	75/90	91/95	88/89	93/95	83/77	84/91	86/94	79/73	93/91	77/92
Using condoms (women age 15–49/men age 15–49) (%)	76/82	79/83	73/82	84/80	84/92	76/85	80/92	79/74	77/84	78/86	58/62	68/90	60/83
Has comprehensive knowledge[6] about HIV (women age 15–49/men age 15–49) (%)	25/33	32/41	19/26	26/21	22/29	36/47	31/43	24/26	20/32	24/23	17/23	31/37	17/35
Knows HIV can be transmitted by breastfeeding (women age 15–49/men age 15–49) (%)	85/78	88/76	83/79	86/71	86/72	88/78	93/77	88/85	86/81	86/78	71/78	90/71	72/79
Knows risk of MTCT can be reduced by mother taking special drugs during pregnancy (women age 15–49/men age 15–49) (%)	50/44	55/49	45/39	46/48	48/63	54/49	35/24	57/41	55/46	63/56	28/29	61/40	47/36

[illegible]

2014 Demographic and Health Survey

Fact Sheet

Ghana

Trends in HIV Testing

Percent of women and men age 15-49 who were ever tested for HIV and received their results

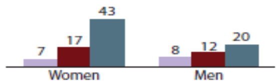

Trends in HIV Prevalence

Percent of women and men age 15-49 who are HIV-positive

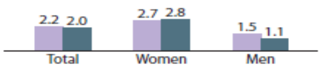

Women's Participation in Household Decisions

Percent of married women age 15-49 who participate in the following decisions either alone or jointly with their husband/partner

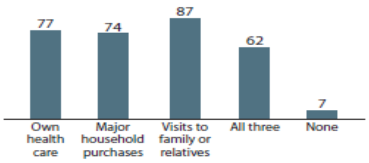

For additional information on the results of the 2014 Ghana Demographic and Health Survey, please contact:

In Ghana:
Ghana Statistical Service (GSS)
P.O. Box GP 1098; Accra, Ghana
Telephone: 233-302-682-661/233-302-663-578
Fax: 233-302-664-301
Email: info@statsghana.gov.gh; www.statsghana.gov.gh

In USA:
The DHS Program
530 Gaither Road, Suite 500; Rockville, MD 20850 USA
Telephone: +1-301-407-6500; Fax: +1-301-407-6501
Email: info@DHSprogram.com; www.DHSprogram.com

The 2014 GDHS was implemented by the Ghana Statistical Service (GSS), the Ghana Health Service (GHS), and the National Public Health and Reference Laboratory (NPHRL) of the GHS. Financial support for the survey was provided by the U.S. Agency for International Development (USAID), the Global Fund through the Ghana AIDS Commission (GAC) and the National Malaria Control Programme (NMCP), the United Nations Children's Fund (UNICEF), the United Nations Development Programme (UNDP), the United Nations Population Fund (UNFPA), the International Labour Organization (ILO), the Danish International Development Agency (DANIDA), and the Government of Ghana. ICF International provided technical assistance through The DHS Program, a USAID-funded project offering support and technical assistance in the implementation of population and health surveys in countries worldwide.

2014 Ghana Demographic and Health Survey (GDHS)

The 2014 Ghana Demographic and Health Survey (GDHS) provides data for monitoring the health situation of the population in Ghana. The 2014 GDHS is the 6th Demographic and Health Survey conducted in the country. The survey is based on a nationally representative sample. It provides estimates at the national and regional levels, as well as for urban and rural areas.

	Number interviewed	Response rate
Households	11,835	99%
Women 15-49	9,396	97%
Men 15-59	4,388	95%

Sanitation and Electricity by Residence

Percent of households

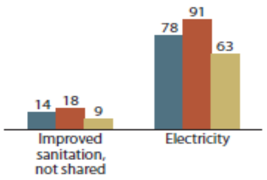

Trends in Fertility

Births per woman for the three-year period before the survey

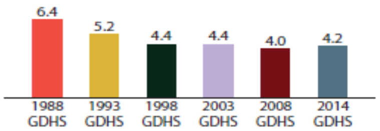

Trends in Family Planning Use

Percent of married women age 15-49 using family planning

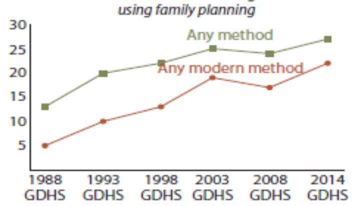

Trends in Childhood Mortality

Deaths per 1,000 live births for the five-year period before the survey

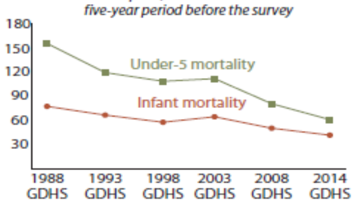

Trends in Maternal Health Care

Percent of live births in the five years before the survey

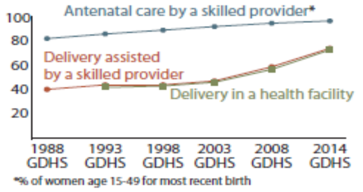

*% of women age 15-49 for most recent birth

Trends in Basic Vaccination Coverage

Percent of children age 12-23 months who received all basic vaccinations

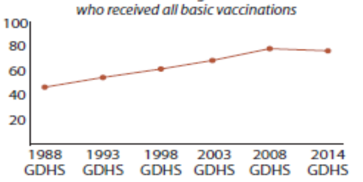

Trends in ITN Use

Percent of children under five and pregnant women age 15-49 who slept under an ITN the night before the survey

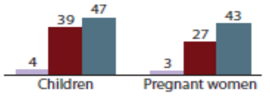

Malaria Prevalence by Region

Percent of children age 6-59 months who tested positive for malaria by microscopy

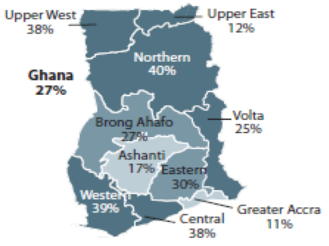

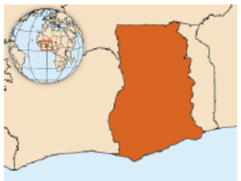

NLiS Country Profile: Ghana

What are the current states of indicators contributing to a comprehensive view of nutrition for health and development in Ghana? See **national** data below.

Child Malnutrition

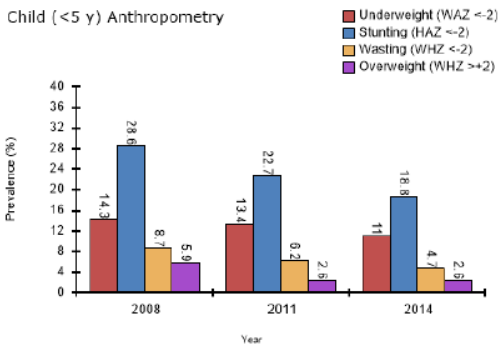

Indicator	Year	Value	Source Info
% Low birthweight (<2500 g)	2011	11.0	View

Malnutrition in Women

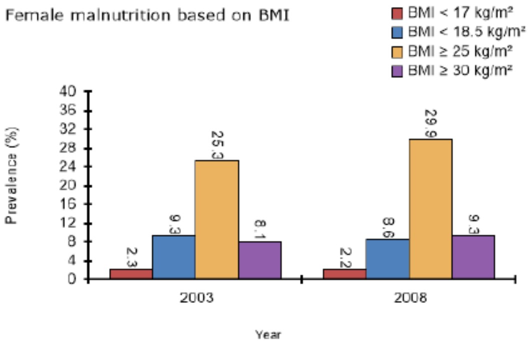

Vitamin and Mineral Deficiencies

Indicator	Year	Value	Source Info
% Anaemia children <5 y (Hb <110 g/L)	2003	76.1	View
% Anaemia pregnant women (Hb <110 g/L)	2003	64.9	View
% Clinical vitamin A deficiency in women(history of night blindness during most recent pregnancy)	2003	7.7	View
% Subclinical vitamin A deficiency in preschool-age children (serum/plasma retinol <0.70 µmol/L)		no data	
Median urinary iodine concentration (µg/L) in children 6-12 y		no data	

Health Services

Indicator	Year	Value	Source Info
% Births attended by skilled health personnel	2011	67.2	View
% Children aged 1 y immunized against measles	2014	92.0	View
% Children with diarrhoea who received zinc		no data	
% Population using an improved sanitation facility	2012	14.0	View
% Population using improved drinking water sources	2012	87.0	View
% Women receiving iron and folate supplements during pregnancy		no data	

Food Security

Indicator	Year	Value	Source Info
% Population below $1 per day	2006	28.6	View
% Population below minimum level of dietary energy consumption	2014-2016	<5.0	View
Iodised salt consumption (% households consuming adequately iodised salt - 15 parts per million or more)	2011	35.0	View

Caring Practices

Indicator	Year	Value	Source Info
% children 0-23 months who were put to the breast within 1 hour of birth	2006	35.2	View
% Infants 6-8 months age who receive solid, semi-solid or soft foods		no data	
% children 6-23 months who receive a minimum acceptable diet		no data	
% Children <5 y with diarrhoea receiving ORT and continued feeding	2011	43.9	View
% Women 15-19 y who are mothers or pregnant with their first child	2008	13.3	View

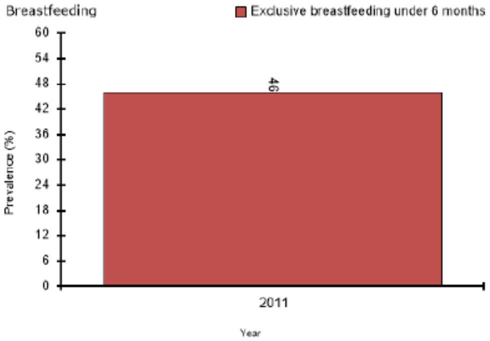

Commitment

Capacity

Indicator	Year	Value	Source Info
General government expenditure on health as % of total government expenditure	2013	10.6	View
Total expenditure on health as % of gross domestic product	2013	5.4	View
Per capita total expenditure on health (US$)	2013	214.0	View
Nutrition component of the United Nations Development Assistance Framework (UNDAF)	2006-2010	Medium	View
Nutrition component of Poverty Reduction Strategy Paper (PRSP)	2006-2009	Weak	View
Nutrition Governance	2009	Weak	View
Maternity leave	2013	12 weeks	View
Monitoring and enforcement of the International Code of Marketing of Breast-milk Substitutes	2007	Yes	View

Indicator	Year	Value	Source Info
Degree training in nutrition exists		no data	
Nutrition is part of medical curricula		no data	
Number of trained nutrition professionals per 100,000 population		no data	
Nursing and midwifery personnel density per 1,000 population	2010	0.9	View
GDP per capita (PPP US$)	2014	4,143	View
GDP per capita annual growth rate (%)	2014	4.2	View
Official development assistance (ODA) received (net disbursements) (% of GNI)	2013	2.8	View
Low-Income Food-Deficit Country (LIFDC)	2015	Yes	View

Meta-indicators

Indicator	Year	Value	Source Info
% Seats held by women in national parliament	2015	10.9	View
Averaged aggregate governance indicators	2013	0.08	View
Gender Inequality Index (GII)	2013	0.534	View
Gender Parity Index in primary level enrolment (ratio of girls to boys)	2014	1	View
Global Hunger Index	2014	7.8	View
Human development index (HDI) value	2013	0.573	View

Female Education Levels

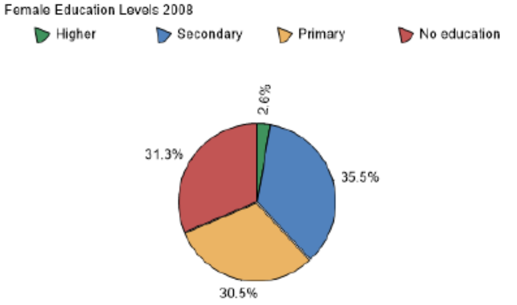

Education

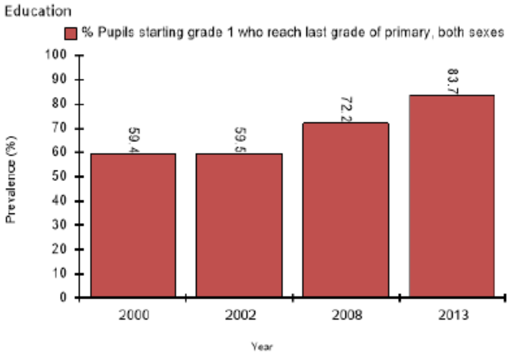

Under 5 Mortality

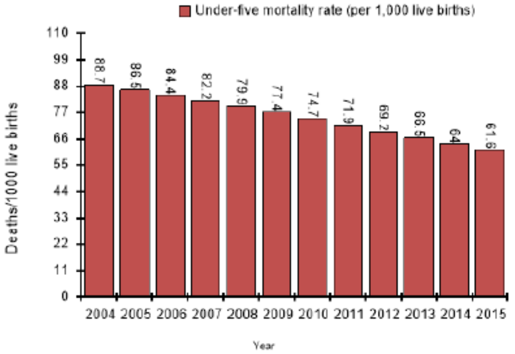

Policies & Actions in the Global database on the Implementation of Nutrition Action (GINA)

Appendix I: Budget

Blood samples	Quantity	Cost/unit (USD)	Amount
Ice Chest for transporting samples (5)	2	60	120
tissue paper (packs)	3	20	60
Needles and syringes (packs)	4	15	60
Hand sanitizer	10	2	20
Gloves (packs)	10	15	150
Transportation of blood samples (trips by flight)	1	100	100
**Subtotal 1**			**510**
**Urine and salt samples**			
30ml cups with screwed tops/urine sample	300	0.5	150
15ml plastic cups for salt with tops	300	0.5	150
Rapid salt testing Kits@50tests	25	10	250
Transportation of urine (by road/day)	1	50	50
**Subtotal 2**			600
Biomarker Analysis			
**Iron**	300	3	900
**Fecal testing**	300	4	1200
**urinary iodine concentration**	24	10	240
**Subtotal 3**			2,440
Office supplies			
Office supplies (printer, scanner and copier)	1	400	400
Accessories(pen, pencils, clip boards, stapler, erasers, etc.) for research assistants	1	50	50
Paper (packs)	10	10	100
**Subtotal 4**			**550**
Communication			
Internet Service (per month)	3	100	300
Modem	1	50	50
**Subtotal 5**			350
Research team			
Token for research participants (mother and child)	150	5	750
Research Assistants per diem (6 people - 20 USD/person)	60	120	7200
Transportation of Research team (each day)	60	25	1500
**Subtotal 6**			9,450
**Demonstration project**			
**Clay containers**	**300**	10	3000
**Cabbage seedlings**			100
**Motorcycle**	**2**	2000	4000
**Fuel and maintenance of motorcycles**		1000	1000

**Miscellaneous**		1000	1000
**Subtotal 7**			10, 000
**GRAND TOTAL**			**23,800**
