# Supplementary material for: Iodine status of reproductive age women and their toddlers in northern Ghana improved through household supply of iodized salt and weekly indigenous meal consumption
Source: PLoS One. 2019 May 31;14(5):e0216931. doi: 10.1371/journal.pone.0216931 (PMC6544231; doi:10.1371/journal.pone.0216931)
Supplement: S3 File — (DOCX) [file pone.0216931.s003.docx]

**Appendix F:**
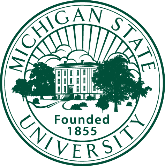
 **Questionnaire**

**Michigan State University**

**Department of Food Science and Human Nutrition**

Community interventions to improve iron and iodine status in mother and child dyads in northern Ghana

**Questionnaire ID………………**

| **Background information** |
| --- |

1. Name of interviewer……………… 2. Date …………… 3. Community ………… 4. Mother’s ID………………

5. Age of mother (yrs) ……………. 6. Weight of Mother (kg)…………... 7. Height of mother (m) …………

8. Mother Hb (g/l) ……… sFe (ug/l) ……….sTfR (nmol/l) ..…… 9. Religion…………. 10. Ethnicity……………….

9. Education: a. none 🞏 b. Primary 🞏 c. Secondary 🞏 d. college/tertiary 🞏

10. Mother’s occupation……………………………. *Mother’s malaria status……………………………..

Marital status: a. married 🞏 b. Single 🞏 c. divorced/separated 🞏

11. Child’s age (months) ………… 12. Child’s height (m) ………… 13. Child’s weight (kg)…………

14. Child’s Hb (g/l) ……………… sFe (ug/l)………. sTfR (nmol/l) ……

Number of siblings…………….. *Child’s malaria status…………….

| **Household characteristics** |
| --- |

15. Sex of household head ………… 16. Age of Household head ………… 17. No of adults in household ……

18. Number of Children <5yrs ………………… 19. Building type ………………… 20. Source of water ………………

21. Type of toilet……………………… 22 Lives with animals in same yard ……..………………..

23. Does your household has any of the under listed items? (Please tick in check box when present)

a. Bicycle 🞏 b. Motorbike 🞏 c. Clock 🞏 d. Radio 🞏 e. Sewing Machine 🞏 f. Bed 🞏 g. Table 🞏

h. Cabinet/Cupboard 🞏 i. Mobile Phone 🞏 j. Refrigerator 🞏 k. Generator/Invertor 🞏 l. Television 🞏

m. Video Deck 🞏 n. Dvd/Vcd 🞏 o. Electricity 🞏 p. Washing Machine 🞏 q. Computer 🞏

r. Digital Camera 🞏 s. Non-Digital Camera 🞏 t. car 🞏 u. tractor 🞏

| 18. Does this household own the under listed (If yes to any, input the corresponding numbers in the 1st column – N*) | N* | 1 | 2 | 3 |
| --- | --- | --- | --- | --- |
| 1. Cattle |  |  |  |  |
| 1. Goat |  |  |  |  |
| 1. Sheep |  |  |  |  |
| 1. Pigs |  |  |  |  |
| 1. Rabits |  |  |  |  |
| 1. Grasscutter |  |  |  |  |
| 1. Chicken |  |  |  |  |
| 1. Guinea fowls |  |  |  |  |
| 1. other poultry |  |  |  |  |
| N* = input number of animals, 1 = none, 2 = more than 95, 3 = don't know | | | | |

| **Women empowerment** |
| --- |

| 19. Who usually | 1 | 2 | 3 |
| --- | --- | --- | --- |
| 1. decides how the money you earn will be used? |  |  |  |
| 1. decides how your husband's/partner's earnings will be used? |  |  |  |
| 1. makes decisions about health care for yourself |  |  |  |
| 1. makes decisions about making major household purchases? |  |  |  |
| 1. makes decisions about making purchases for daily household needs? |  |  |  |
| 1. makes decisions about visits to your family or relatives? |  |  |  |
| 1. makes decisions about how many children to have? |  |  |  |
| Key: 1 = you, 2 = partner, 3 = both | | | |

20. Who earns more money in your household? a. you 🞏 b. partner 🞏 c. about same 🞏

**24 HR Recall Questionnaire**

**Option A**

| I would like to ask you about liquids or foods that (NAME……...)/you may have had yesterday during the day or at night. I am interested in whether your child/you had the item even if it was combined with other foods. | | | | | | | | | | |
| --- | --- | --- | --- | --- | --- | --- | --- | --- | --- | --- |
| Did (NAME ………...)/you drink (eat) | Child | | | | | Mother | | | | |
|  | Yes | No | DK | Freq | Qty (g) | Yes | No | DK | Freq | Qty (g) |
| 1. Milk such as tinned, powdered, or fresh animal milk? |  |  |  |  |  |  |  |  |  |  |
| 1. Tea or coffee? |  |  |  |  |  |  |  |  |  |  |
| 1. Any other liquids (juice, cocoa)? |  |  |  |  |  |  |  |  |  |  |
| 1. Bread, rice, noodles, or other foods made from grains (kenkey, banku, koko,tuo zaafi, akple, weanimix)? |  |  |  |  |  |  |  |  |  |  |
| 1. Pumpkin, red or yellow yams, carrots, sweet potatoes that are yellow or orange inside? |  |  |  |  |  |  |  |  |  |  |
| 1. White potatoes, white yams, manioc, cassava, cocoyam, fufu or any other foods made from roots, tubers or plantain? |  |  |  |  |  |  |  |  |  |  |
| 1. Any dark green, leafy vegetables (kontomire, aleefu, ayoyo, kale, cassava leaves)? |  |  |  |  |  |  |  |  |  |  |
| 1. Ripe mangoes, pawpaw? |  |  |  |  |  |  |  |  |  |  |
| 1. Any other fruits or vegetables (e.g. bananas, avocados, tomatoes, oranges, apples)? |  |  |  |  |  |  |  |  |  |  |
| 1. Liver, kidney, heart or other organ meats? |  |  |  |  |  |  |  |  |  |  |
| 1. Any meat, such as beef, pork, lamb, goat, chicken, or duck? |  |  |  |  |  |  |  |  |  |  |
| 1. Eggs? |  |  |  |  |  |  |  |  |  |  |
| 1. Fresh or dried fish or shellfish (e.g. prawn, lobster)? |  |  |  |  |  |  |  |  |  |  |
| 1. Any foods made from beans, peas, lentils, or nuts? |  |  |  |  |  |  |  |  |  |  |
| 1. Cheese, yogurt or other milk products? |  |  |  |  |  |  |  |  |  |  |
| 1. Any oil, fats, or butter, or foods made with any of these? |  |  |  |  |  |  |  |  |  |  |
| 1. Any sugary foods such as chocolates, sweets, candies, pastries, cakes, or biscuits? |  |  |  |  |  |  |  |  |  |  |

***Option A* procedure:** Ask for what has been consumed the previous day without mentioning food items to avoid prejudice. Tick as foods are mentioned, probe for frequency and usual quantities in the past 7 days. Probe for other foods consumed within the week, their frequencies and usual quantities consumed. Probe for alcohol consumption, smoking and substance abuse

**Option B**

| **24 hour recall Day of the week: ……………………………………………….** | | | |
| --- | --- | --- | --- |
| **Time / period of day** | **quantity of food eaten** | **Detailed food description** | **Source of food** |
|  |  |  |  |
|  |  |  |  |
|  |  |  |  |
|  |  |  |  |
|  |  |  |  |
|  |  |  |  |
|  |  |  |  |
|  |  |  |  |

**Probe for alcohol consumption, smoking and substance abuse**
